# Heat-Up Colloidal Synthesis of Shape-Controlled Cu-Se-S Nanostructures—Role of Precursor and Surfactant Reactivity and Performance in N_2_ Electroreduction

**DOI:** 10.3390/nano11123369

**Published:** 2021-12-12

**Authors:** Stefanos Mourdikoudis, George Antonaropoulos, Nikolas Antonatos, Marcos Rosado, Liudmyla Storozhuk, Mari Takahashi, Shinya Maenosono, Jan Luxa, Zdeněk Sofer, Belén Ballesteros, Nguyen Thi Kim Thanh, Alexandros Lappas

**Affiliations:** 1 Biophysics Group, Department of Physics and Astronomy, University College London, London WC1E 6BT, UK; l.storozhuk@ucl.ac.uk; 2 UCL Healthcare Biomagnetics and Nanomaterials Laboratories, 21 Albemarle Street, London W1S 4BS, UK; 3 Department of Inorganic Chemistry, University of Chemistry and Technology Prague, Technicka 5, 16628 Prague, Czech Republic; nikolaoa@vscht.cz (N.A.); jan.luxa@vscht.cz (J.L.); 4 Institute of Electronic Structure and Laser, Foundation for Research and Technology-Hellas, Vassilika Vouton, 71110 Heraklion, Greece; ganton@iesl.forth.gr; 5 Department of Chemistry, University of Crete, Voutes, 71003 Heraklion, Greece; 6 Catalan Institute of Nanoscience and Nanotechnology (ICN2), CSIC and the Barcelona Institute of Science and Technology, Campus UAB, Bellaterra, 08193 Barcelona, Spain; marcos.rosado@icn2.cat; 7 School of Materials Science, Japan Advanced Institute of Science and Technology, 1-1 Asahidai, Nomi 923-1292, Ishikawa, Japan; mari@jaist.ac.jp (M.T.); shinya@jaist.ac.jp (S.M.)

**Keywords:** bottom-up synthesis, electrochemistry, wet chemistry, morphology, copper chalcogenides, nitrogen reduction reaction, metal-organic chemistry

## Abstract

Copper selenide-sulfide nanostructures were synthesized using metal-organic chemical routes in the presence of Cu- and Se-precursors as well as S-containing compounds. Our goal was first to examine if the initial Cu/Se 1:1 molar proportion in the starting reagents would always lead to equiatomic composition in the final product, depending on other synthesis parameters which affect the reagents reactivity. Such reaction conditions were the types of precursors, surfactants and other reagents, as well as the synthesis temperature. The use of ‘hot-injection’ processes was avoided, focusing on ‘non-injection’ ones; that is, only heat-up protocols were employed, which have the advantage of simple operation and scalability. All reagents were mixed at room temperature followed by further heating to a selected high temperature. It was found that for samples with particles of bigger size and anisotropic shape the CuSe composition was favored, whereas particles with smaller size and spherical shape possessed a Cu_2−x_Se phase, especially when no sulfur was present. Apart from elemental Se, Al_2_Se_3_ was used as an efficient selenium source for the first time for the acquisition of copper selenide nanostructures. The use of dodecanethiol in the presence of trioctylphosphine and elemental Se promoted the incorporation of sulfur in the materials crystal lattice, leading to Cu-Se-S compositions. A variety of techniques were used to characterize the formed nanomaterials such as XRD, TEM, HRTEM, STEM-EDX, AFM and UV-Vis-NIR. Promising results, especially for thin anisotropic nanoplates for use as electrocatalysts in nitrogen reduction reaction (NRR), were obtained.

## 1. Introduction

Noble metal nanoparticles (NPs) have been shown to possess interesting optical properties. More recently, non-noble element copper-deficient copper chalcogenide nanostructures have also been proven to display remarkable plasmonic features. For instance, if colloidal chemical synthetic routes are used, the precursor ratio at the copper-tellurium system has been reported to affect the final Cu:Te proportion in the nanomaterial product. This, consequently, can influence the resulting crystal structure [1]. Copper selenide NPs are p-type semiconductors which can find applications in several domains, including energy conversion [2] and storage as well as biomedicine [3]. For example, the Cu_2−x_Se phase has a direct band gap of 2.1 eV. The wide range of possible band gap energies and varying electronic behaviours are most probably related to the variations in Cu to Se stoichiometry, recombination sites due to dislocations, size effects and/or the oxidation state of Cu and Se. Thus, it is clear that the optoelectronic properties of this system are promising and potentially tunable due to the broad range of possible crystal structures, particle sizes and respective band gap values [4]. More specifically, such applications have to do with Li-ion batteries, solar cells and photothermal therapy [5,6]. Several stoichiometric forms of copper selenides exist, such as Cu_2_Se, Cu_3_Se_2_, CuSe, CuSe_2_, Cu_5_Se_4_, Cu_7_Se_5_ and non-stoichiometric Cu_2−x_Se. Their crystalline structures can be cubic, hexagonal, tetragonal, orthorhombic and monoclinic [7]. Several works have highlighted the importance of the choice of the surface ligands in order to tailor smoothly the final optical properties which are crucial for the relative applications. For example, Balitskii et al. illustrated that ligands with electron trapping or donating functional groups could influence the free carrier density and, therefore, the resulting localized surface plasmon resonances (LSPRs) [8]. Zhu et al. synthesized Cu_2−x_Se with a cubic phase and showed that by modifying the oleic acid to oleylamine ratio, the LSPR wavelength could be tuned over a wide range between 1030–1260 nm [9].

Although there are lots of efficient synthesis routes for copper selenide and other types of metal chalcogenide nanostructures, including solvothermal and colloidal chemical methods [10,11], or even different approaches such as metal-organic vapor phase epitaxy [12], phase-controlled synthesis of Cu-Se nanostructures with high yield and by avoiding the so called ‘hot-injection’ approaches is still under development [13]. Other synthetic methods include, for example, the sacrificing template method [14], hydrothermal synthesis and aerosol-assisted chemical vapor deposition [15]. The morphologies of the copper selenide nanoscale system span from spherical NPs, nanoboxes, nanosheets, nanoribbons, nanocubes and hexagonal nanoplates to other configurations [14]. Especially regarding the Cu_2−x_Se composition, there are fewer reports on the tuning of their LSPR absorbance over a wide range, in comparison with the Cu_2−x_S NP system. This may be due to the difficulty to prepare high quality, water soluble and biocompatible copper selenide nanostructures. In fact, the relatively low mutual affinity of copper and selenium may make the size or composition control of the Cu-Se system complicated, especially compared with the Cu-S one [16]. The Cu vacancies (hole densities) in copper chalcogenide NPs arise from the release of copper ions from their surface or grain boundaries. Increasing the number of Cu vacancies, by modifying the value of ‘x’, affects not only the optical properties but can also lead to the improvement of the conductivity of copper chalcogenide NPs [17]. Similarly, decreasing ‘x’ is associated with a decrease in free hole concentration [16]. A lower value of ‘x’ is associated with higher proportion of Cu, also taking into account the Cu_2−x_Se formula.

Nanostructures with mixed compositions such as copper sulfide-selenide and copper telluride-sulfide prepared by scalable non-injection routes have attracted interest since their optical properties are quite similar to those of the pure binary copper chalcogenides and they can be tuned in a wide range in the near-infrared (NIR) region [18]. In fact, heat-up approaches in organic medium have been shown to be efficient also for the production of other types of ternary systems, such as In-As-Sb semiconductor quantum dots which displayed controlled optoelectronic properties [19]. Considering the composition-dependent optical/electrical properties of metal chalcogenides, in the present work, a one-pot, heat-up metal-organic chemical method was developed, aiming to obtain Cu_x_S_y_Se_z_ nanostructures in a range of compositions, morphologies and crystal structures. Moderate temperatures were employed and Al_2_Se_3_ was proven to act as an effective selenium source in an unprecedented manner. Dodecanethiol, on the other hand, was shown to act as a massive sulfur source, which was incorporated in the resulting nanostructures under certain reaction conditions. LSPR peaks were observed in a wide range in the NIR, depending on the features of the different samples. Considerable electrocatalytic activity in the nitrogen reduction reaction was interestingly recorded for some of the copper chalcogenide nanostructures of the present study. This reaction constitutes a mildly operating and environmentally friendly way to produce NH_3_ from N_2_ [20], and our results are discussed on the basis of the distinct morphologies, compositions and band gaps of different samples.

## 2. Results and Discussion

### 2.1. Synthesis, Composition and Morphology

**Figure 1:**
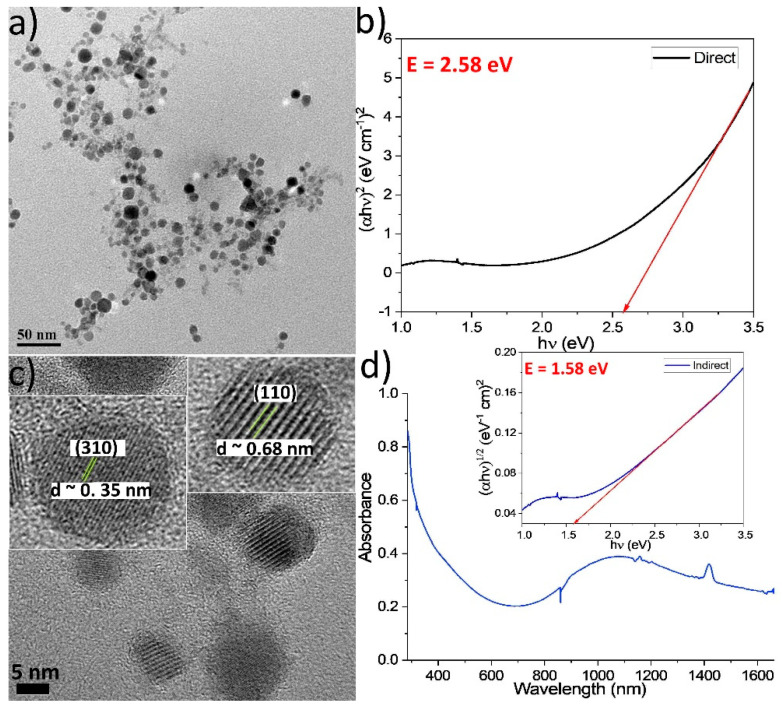
TEM image (**a**), Tauc plot for direct transitions (**b**), HRTEM image with manually magnified NPs as insets (**c**) and UV-Vis-NIR spectrum (**d**) with Tauc plot for indirect transitions (inset) for sample Sa1.

Initially our goal was to create copper selenide nanostructures using a simple so-called ‘heat-up’ approach where all reagents are mixed at room temperature and the obtained mixture is heated to a certain temperature. The stoichiometric (1:1) ratio between Cu and Se precursors was kept constant during all synthetic endeavours. We were inspired from the work by Xiao et al. [21] who showed that in principle it is possible to avoid the hot-injection synthetic protocols, which cannot be easily scaled up. A new precursor was tested, aluminium selenide (Al_2_Se_3_), which had not been previously used to prepare copper selenide NPs. In addition, the final reaction temperature was moderately high (200 °C) in most of the samples produced. In the aforementioned paper [21], SeC(NH_2_)_2_ and elemental Se were employed as selenium sources. With our method, a light green colour was observed at the colloidal dispersion of the NPs produced (Figure S1a), giving a first suggestion of their composition, implying a successful semiconductor production. Apart from Al_2_Se_3_, Cu(acac)_2_ was used as the source of copper (see also Table S1 at the Supp. Info, which lists the reagents used for all samples of the current study). Indeed, TEM images for sample Sa1 show the presence of nearly spherical NPs with an average size of 10 ± 2.3 nm (Figure 1a). It is suggested that the pair of surfactants used, oleic acid and oleylamine (Oam), which has been widely employed in NP synthesis [22], helped to keep a controlled size level, hindering uncontrolled growth and aggregation phenomena. Still, the presence of lithium amide seemed not enough to induce an anisotropic plate-like morphology, unlike the case of our previous work on Cu_1.25_Te NPs [1]. Figure 1c shows high resolution-TEM (HRTEM) images of Sa1. The existence of the (110) plane of the orthorhombic Cu_2_Se phase (ICDD No 00-019-0401) is indicated by the wide lattice spacings (~0.68 nm) in some NPs (Figure 1c, inset on the right side). Such unusually large periodicity has been observed also in a work by White et al., where it was reported to be related to the Cu^+^ vacancy ordering mode [23]. Other NPs have apparently shorter lattice spacings (~0.35 nm), implying the presence of the (310) plane. ICP-AES (Inductive coupled plasma atomic emission spectroscopy) measurements revealed an elemental ratio Cu:Se:Al = 5.7:1:0.2. The excess of Cu probably occurs in the form of amorphous copper oxide surrounding the Cu-Se cores. The crystal structure of copper selenide might hinder the insertion of ‘foreign’ elements into its crystal lattice, thus not allowing the presence of a significant amount of aluminium in it [24]. Regarding the fate of the Al of the precursor molecule, it is supposed that it can form some complex compounds with the excess surfactants: afterwards, it can be removed either in the form of a complex or as unreacted Al as part of the supernatant during the centrifugation cycles. Unfortunately, the relatively low yield for Sa1 did not allow to isolate sufficient amount of solid powder for XRD measurements. The UV-Vis-NIR spectrum for this sample features a peak at around 1080 nm (Figure 1d). In a previous report, Cu_1.81_Se NPs of 15 nm size displayed a LSPR band centered at 1100 nm [25]. However, Kriegel et al. had observed the LSPR band with a maximum at around 1350 nm for 12 nm Cu_1.8_Se NPs [26]. In another work, 6 nm Cu_2−x_Se NPs exhibited the highest energy LSPR peak at about 1000 nm, with a LSPR peak near 1300 nm arising for NPs of a 13 nm size [16]. A long and overly comprehensive review paper by Coughlan et al. contains much information on the physical properties of copper chalcogenide NPs, including Cu-Se ones [27]. The respective Tauc plots and the derived band gaps of the different samples of the current study are discussed below.

**Figure 2:**
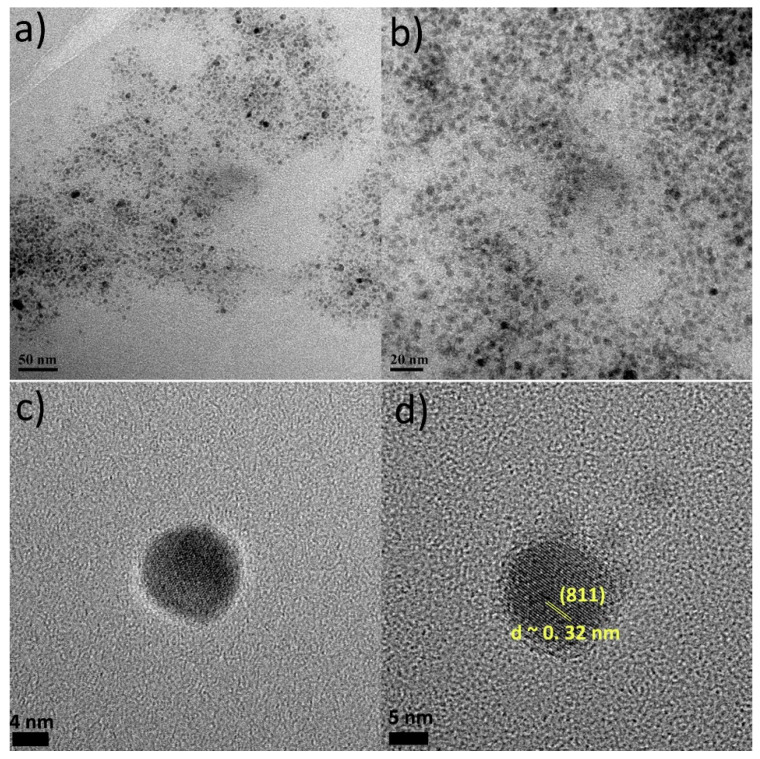
TEM images (**a**,**b**) and HRTEM images (**c**,**d**) of Sa2.

For the synthesis of Sa2 we attempted to use a more ‘direct’ selenium source, which has also been proven to act successfully in the preparation of metal-selenium chalcogenide nanostructures, that is, elemental Se. Elemental selenium and tellurium are insoluble in the majority of known solvents, which complicates the synthesis of nanostructures containing those elements using ‘simple’ colloidal chemical routes [28]. In our previous work on CuTe nanoplates, trioctylphosphine (TOP) first reacted with Te to provide the TOP-Te precursor [1]. Since selenium has been reported to be soluble in a dodecanethiol-oleylamine mixture [29], we combined these two reagents in sample Sa2. In addition, we added TOP, aiming to study if its co-existence with Li-amide would facilitate to obtain an anisotropic shape, such as in the case of our previous work on Cu-Te nanostructures [1]. The reaction ran rather smoothly, and some yellow colour arose in the course of the synthesis, possibly implying the presence of intermediate complex compounds. This was followed by the appearance of dark brown colour for the final colloid. In fact, TEM images for Sa2 at Figure 2a,b showed the presence of relatively small spherical NPs with a size of 4.5 ± 0.6 nm. The observed size and shape of the NPs cannot be regarded as an unexpected surprise: first of all, TOP was used in its pure form and not in the form of a TOP-Se compound, for instance, such as in the case of our Cu-Te work. Essentially, TOP is a voluminous molecule that has been often associated with hindering excessive particle growth, thus keeping small particle sizes [30]. Another difference with the protocol we used for the Cu-Te is the presence of dodecanethiol in the sample under discussion. Ιnterestingly, ICP-AES measurements revealed a ratio Cu:S:Se = 3.2:1:0.32 in Sa2. It seems that dodecanethiol acted as sulfur donor, whereas a variety of stable complex compounds may have been formed, hindering the incorporation of Se in the NPs. HRTEM images at Figure 2c,d confirm the spherical shape of these NPs, indicating a lattice spacing of around 0.32 nm, assigned to the (811) plane of the Cu_31_S_16_ monoclinic crystal structure (ICDD 00-034-0660), but it could also potentially match with the (111) plane of the cubic Cu_1.8_Se phase (ICDD 01-088-2045). Additionally, in this sample, the presence of a portion of amorphous copper oxide cannot be excluded, since the samples were kept in air after synthesis. It has to be noted that the reactivity of Cu(Oac)_2_ (used in Sa2 as copper precursor) is quite lower than that of Cu(acac)_2_ (the Cu source in Sa1, see also experimental part and Table S1 at the Supplementary Materials). Precursors with higher reactivity tend to induce isotropic growth [21]. Still, in the case of Sa2 the reaction conditions used were sufficient to maintain small size and isotropic growth. A peak at around 1360 nm at the UV-Vis-NIR spectrum (Figure S3 at the Supplementary Materials) of Sa2 is in agreement with the presence of copper sulfide composition. The copper sulfide semiconductor shows a prominent LSPR peak in the second biological spectral window of the NIR region (1000–1350 nm), unlike plasmonic metals Ag, Au and Cu which show LSPR peaks in the visible range [31]. Though the particles of the Sa2 sample are relatively small, it is possible that a ‘limited aggregation’ or a somewhat dense arrangement of the NPs led to a red shift of the band to 1360 nm [32]. Normally, smaller NP sizes exhibit LSPR peaks at lower wavelengths in comparison with bigger NPs [16]. Unfortunately, complicated washing procedures resulted in the loss of a significant number of particles, hindering the acquisition of a sufficient sample quantity for XRD measurements.

**Figure 3:**
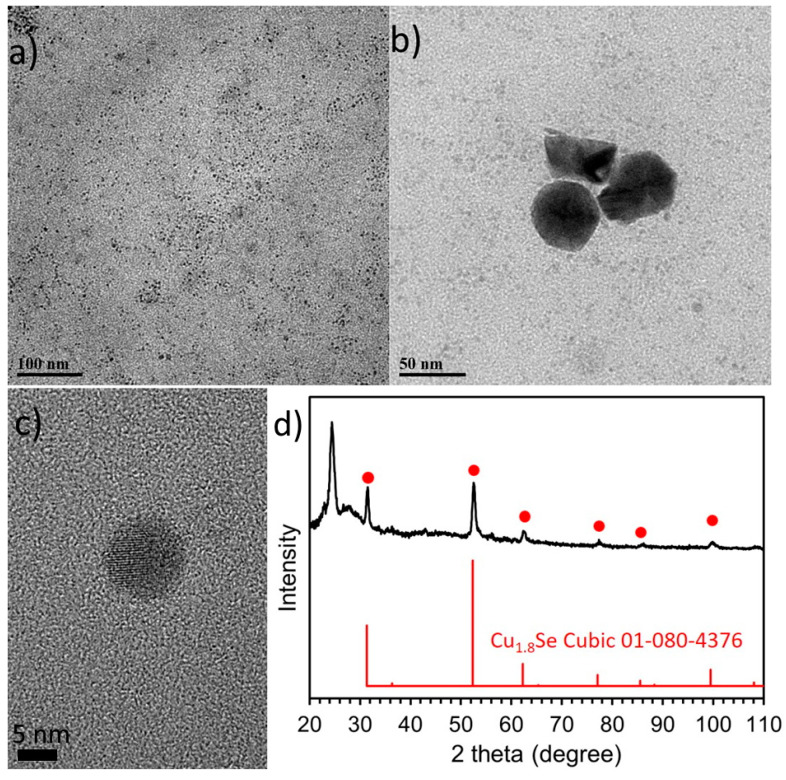
TEM images (**a**,**b**), HRTEM image (**c**) and XRD measurement (**d**) of Sa3.

For the sample Sa3, having observed that we successfully produced NPs using the protocols for Sa1 and Sa2, we decided to test a new protocol by combining those two ones. More specifically, since Al_2_Se_3_ seemed to be able to indeed act as the Se precursor, we replaced the Se elemental precursor of Sa2 with Al_2_Se_3_, which we used in the synthesis of Sa1. Figure 3a,b demonstrate that the majority of the produced NPs had an average size of 4.0±0.5 nm, though a small population of bigger NPs of around 35–45 nm in size was also spotted. It seems that the presence of TOP still maintained most of the NPs at a small size, but the distinct decomposition mode of Al_2_Se_3_ possibly allowed to produce a portion of larger NPs too. HRTEM images show that some NPs contain crystalline domains which are not always clearly distinguishable, and this complicates the determination of lattice spacing. Still, the NP depicted at Figure 3c has two rather clearly observed crystalline domains, with lattice spacings 0.33 and 0.2 nm, assigned to the (111) and (220) planes, respectively, of the Cu_2_Se cubic crystal structure (ICDD 03-065-2982). The ICP-AES measurements of Sa3 showed a ratio of Cu:Se:S:Al = 2.9:1:0.4:0.4. It seems that also in this sample, Al_2_Se_3_ acted as an effective selenium source. The partial incorporation of sulfur (due to the dodecanethiol (DDT)) and of Al is also noticed. It seems that under different synthetic conditions (samples Sa1 and Sa3) Al_2_Se_3_ remains an efficient precursor for the NP system under discussion. This may be due to the fact that copper has low solubility in aluminium at low temperatures, thus preventing the massive insertion of Al in the NPs’ crystal lattice [33]. Even at room temperature, a solid solution between Cu and Al might be metastable or difficult to retain, thus suggesting why Al was not massively incorporated in the copper selenide nanostructures. The XRD measurement of Sa3 (Figure 3d) is in fair agreement with HRTEM, indicating the existence of the fcc Cu_1.8_Se phase, as the peaks at 52.8, 31.5 and 62.8^o^ match the ICDD 01-080-4376 pattern of copper selenide (for Co Ka source XRD). Since ICP measurements revealed an abundance of Cu, the presence of amorphous copper oxides surrounding the Cu-Se core cannot be excluded. The UV-Vis-NIR spectrum recorded for Sa3 (Figure S4 in the Supporting Information) does not permit to deduce some clear insights, possibly because of the relative particle size polydispersity (co-existence of small with bigger particles) and the presence of Al impurities.

As mentioned above, Cu(acac)_2_ has higher reactivity than Cu(OAc)_2_; therefore, at sample Sa4 we replaced the latter precursor by the acetylacetonate one, to test its effect, keeping all the other reaction conditions the same as in the case of Sa2. Apart from the fact that precursors with higher reactivity are expected to induce isotropic growth more easily, they tend to produce NPs of a smaller size [21]. In fact, TEM images of Sa4 at Figure S5 show that it consists of NPs of a size of about 11.2 ± 2.4 nm, which is somewhat bigger than that of Sa2, whereas a random appearance of a small population of larger NPs in a few zones of the TEM grid was also spotted. This finding demonstrates that the reactivity of a precursor, in this case the Cu(acac)_2_, is not the sole factor which determines the fate of the particle growth. Both precursors, of copper and selenium may not only interact with each other but also with the variety of the other reagents in the flask: all these molecules with amine, carboxylic acid, amide, thiol and phosphine functional groups present distinct functionalities and it is hard to predict how they will behave when they are mixed altogether with the two precursors and heated at high temperature. In our previous work with CuTe NPs, we had noticed that indeed the use of Cu(acac)_2_ instead of Cu(OAc)_2_ resulted in smaller NP size: in particular, two populations of rectangular particles with 22.4 and 12.2 nm average sizes were obtained with the former precursor, whereas the Cu(OAc)_2_ systematically yielded a single NP population with an average size above 30 nm [1]. Possibly the DDT, present at Sa4, interacted with the Cu(acac)_2_ through its thiol moiety in a manner that hindered the tendency of this copper salt to produce smaller NP sizes. A broad, although not well-defined, band at around 1300 nm (Figure S6) of wavelength was recorded for Sa4, which could imply the existence of a copper sulfide-rich composition, since the Cu_31_S_16_ sample Sa2 showed rather similar optical properties with the Sa4 [34]. The colloidal dispersion of Sa4 had an orange-like colour (Figure S1b).

**Figure 4:**
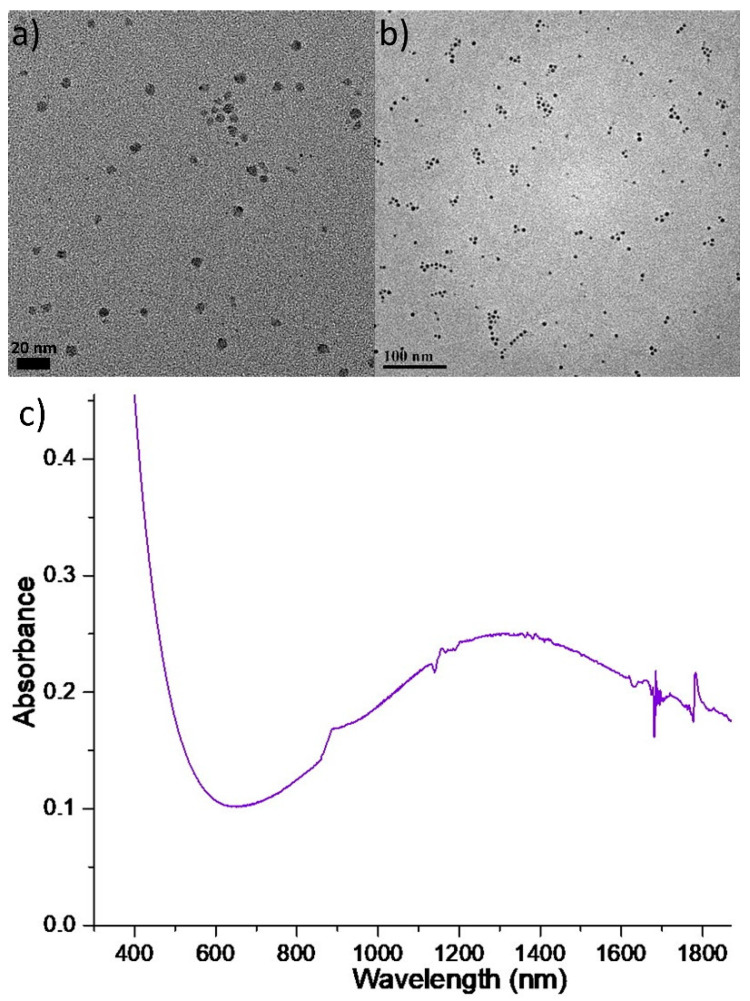
TEM images (**a**,**b**), and UV-Vis-NIR spectrum (**c**) of Sa5.

Aiming to increase the relatively low yield of Sa4, at sample Sa5 we applied a somewhat higher reaction temperature, with a longer reaction time. Indeed the yield was a bit higher than that of Sa4, and a brownish colloidal product was observed for Sa5. TEM images at Figure 4a,b show the presence of nearly spherical NPs with a mean size of 5.2 ± 0.4 nm. It is possible that the higher reaction temperature (220 °C instead of 200 °C) increased the precursors’ reactivity and accelerated the diffusion between copper and selenium, resulting in smaller NP size than in Sa4 [21]. The production of a big amount of nuclei in a short time, due to the higher reaction temperature, may also explain the acquisition of smaller NPs: not much available material would be left to consume and keep feeding the particles, thus an extended stage of particle growth would not be easy to occur. Unfortunately the XRD pattern for Sa5 (Figure S7) does not allow to derive a safe idea on the composition of these particles. The combination of peaks at 24.3° and 45° cannot be matched to a known composition with certainty. ICP-AES composition and STEM-EDS analyses, however, indicate the presence of the copper sulfide phase. In particular, ICP gave a Cu:S:Se ratio of 2.5:1:0.13. In this sample, Se seems to be present in very minor concentrations compared with sulfur. HRTEM images for this sample (Figure S8) confirm that particles are almost spherical in shape and lattice spacing of around 0.3 nm is measured in some of them; however, the mixed composition of Sa5 hinders a safe assignment of a specific crystal structure for this lattice plane. STEM-EDS elemental mapping analysis (Figure S9) yielded a Cu:S ratio of around 1.8 and demonstrated the homogeneous distribution of all elements throughout the NP volume. It seems that under the synthetic conditions used for Sa5, DDT was confirmed to act as a massive sulfur source [18,35]. Concerning the UV-Vis-NIR spectrum of Sa5 (Figure 4c), the LSPR band centered at approximately 1300 nm is in accordance with the existence of a Cu_2−x_S phase [18].

At the sample Sa6 we decided to study the role of another reagent which has been widely used in nanoparticle synthesis, including for metal chalcogenide systems, that is, trioctyphosphine oxide (TOPO). Therefore, in sample Sa6 we followed the synthetic protocol used for the Sa2, with the exception of replacing the TOP by TOPO. The latter reagent can act as a surfactant, solvent and capping agent, allowing reactions to be carried out also at very high temperatures, exceeding 300 °C. The steric properties of its alkyl groups influence particle growth, tailoring particles morphology [36]. For example, in the case of TOPO-capped CdSe, the TOPO binds to the surface cadmium sites through the lone pairs of electrons on the phosphine oxide group, forming dative bonds [37,38]. In particular, the use of technical-grade (not highly pure) TOPO has been found to be more suitable than the pure one for production of anisotropic nanostructures. The large amount of impurities strongly coordinated to the precursor ions result in a slower growth speed. Τhe role of impurities can sometimes be responsible for difficulties to reproduce a certain nanomaterial, but at times it can also be beneficial, in what concerns a controlled colloidal synthesis aiming to achieve sols with desired features [39,40]. The impurities of technical-grade TOPO, which was used in Sa6, have been quite well investigated in the current stage and also identified via techniques such as ^31^P NMR [39].

**Figure 5:**
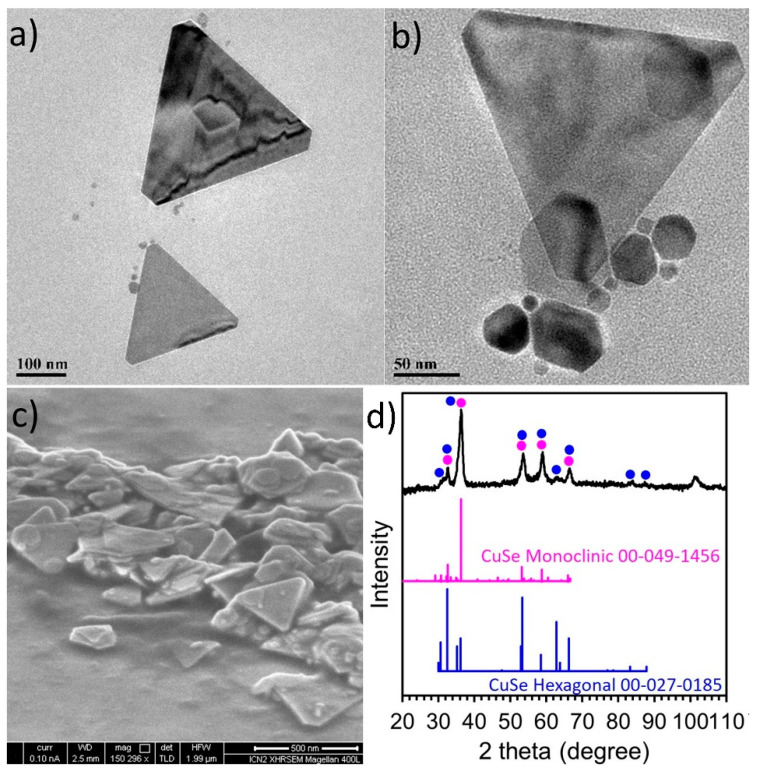
TEM images of sample Sa6 (**a**,**b**), SEM image (**c**) and XRD measurement (**d**).

**Figure 6:**
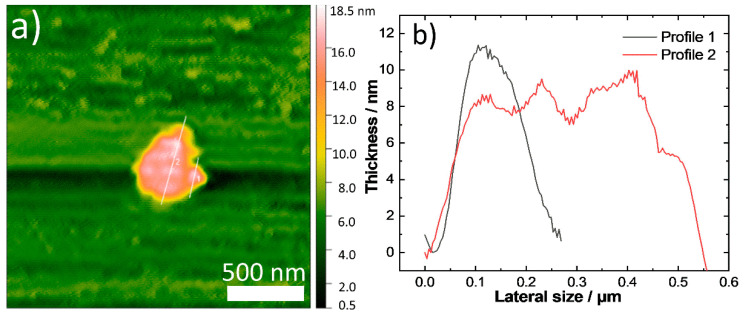
AFM image of a thin nanoplate (**a**) of sample Sa6 together with the respective measured height profile (**b**).

Depending on the overall growth rate of the reaction, a prefered growth mode along a particular axis of the nanostructures can take place, resulting in anisotropic shapes [41]. With all the above in mind, we carried out the synthesis of Sa6 in the presence of technical-grade (90% purity) TOPO and indeed we observed an anisotropic growth mode, contrary to the previous samples of the current study. TEM and SEM images of Figure 5 depict a tendency for the formation of trigonal nanoplates with sizes of 200–250 nm, with a thickness of around 10–13 nm, as revealed by AFM, HAADF-STEM and SEM imaging (Figure 6 and Figure S10 bottom). The determination of the height profile by AFM was manageable by spotting a larger spheroid particle with a 10 nm thickness and a 550 nm lateral size. A lower population of smaller, also anisotropic, particles was spotted, too. The STEM-EDX analysis of this sample (Figure S10) indicates that there is considerable presence of both Cu and Se throughout the volume of the particles with a rather small concentration of sulfur. Interestingly, the XRD measurement for the sample S6 (Figure 5d) illustrates that the main component is the monoclinic and stoichiometric CuSe phase, as the peaks at 32.6, 36, 53 and 59°, are in agreement with the ICDD pattern 00-049-1456. A small portion of the stoichiometric CuSe hexagonal phase (ICDD pattern: 00-027-0185) cannot be excluded. ICP-AES also indicate an approximately equiatomic ratio between copper and selenium elements in the sample under discussion (Cu:Se:S = 1.2:1:0.1). Therefore, all measurements for this sample denote, rather surprisingly, that the presence of DDT was not able to lead to a significant insertion of sulfur in the particle volume. It has been reported that a lower reactivity among the reagents in the reaction pot lowers the chemical potential but can facilitate the acquisition of larger CuSe particles with a Cu to Se ratio of 1:1 [21]. This is in line with what we observed in the sample under study. In these conditions, the mutual diffusion between copper and selenium becomes slower, nucleation rate also slows down and anisotropic growth is favored, whereas the formation of the nonstoichiometric Cu_2−x_Se phase is not prefered. Τhe colloidal dispersion of the stoichiometric CuSe nanostructures of sample Sa6 was dark brown, darker than the one of the previous Cu_2−x_Se samples, and its UV-Vis-NIR spectrum (Figure S11 at the Supplementary Materials) did not contain any clear band within the measured range of wavelengths, probably because of the very large size of these structures. Compared with sample Sa2, which was Se-deficient, the Sa6 contained the ‘expected’ proportion of Se based on the starting reagent concentrations. Possibly some amount of Se at sample Sa2 formed a complex with TOP, resulting in not fully decomposed TOP-Se. TOPO, in Sa6 is already oxidized and could not form a complex with selenium, thus allowing Se to be easily blended with Cu.

**Figure 7:**
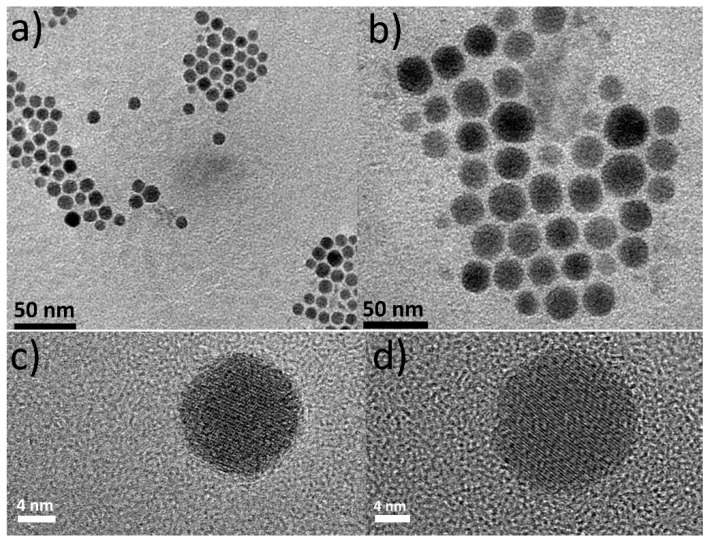
TEM images (**a**,**b**) and HRTEM images (**c**,**d**) of sample Sa7.

**Figure 8:**
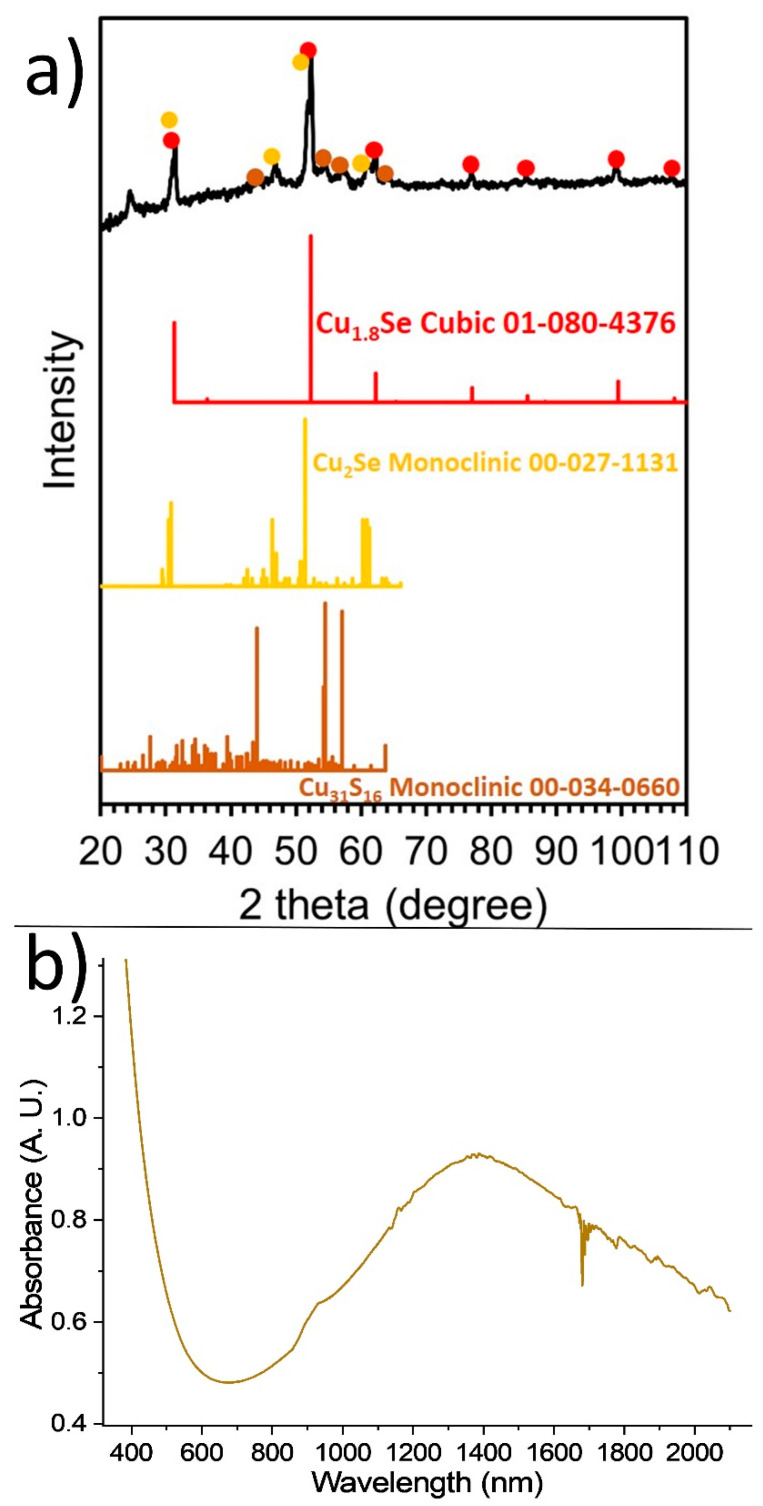
XRD measurement (**a**) and UV-Vis-NIR spectrum (**b**) of Sa7.

Inspired by a protocol published by Lesnyak and co-workers [18], at sample Sa7 we used a mixture of excess DDT and OAc (in 1:2 volume ratio) together with a copper salt (Cu(OAc)_2_ in our case) and elemental Se. Experimental observations confirmed that the aforementioned mixture also allowed the Se to be dissolved quite well. The above-mentioned reagents TOP, OAm and Li-amide were used again as in the previous samples (Sa1–Sa5). Figure 7a,b shows the presence of nearly spherical particles with a size of 8.9 ± 1.1 nm. Though we used more chemical reagents than those of the protocol by Saldanha et al. [18], this particle size was not much different than that of their Cu_2−x_S, Cu_2−x_Se_y_S_1−y_ and Cu_2−x_Te_y_S_1−y_ NPs. A fair amount of the particles of Sa7 were monocrystalline, with the HRTEM image of Figure 7d revealing a lattice spacing distance *d* of around 0.20 nm, implying the presence of the (10 5 2) plane of the cubic Cu_31_S_16_ crystal structure but it could also be assigned to the (220) lattice plane of the cubic Cu_1.8_Se (ICDD 01-088-2045). The XRD measurement for sample Sa7 (Figure 8a), indicated that in this sample, copper selenide phases (especially the Cu_1.8_Se one) seem to be in excess, with an amount of Cu_31_S_16_ phase being present, too. Essentially, ICP yielded a Cu:S ratio of 2.3 and STEM-EDX provided a ratio of 2.4 (Figure S12). A slight excess of copper present in the latter measurements might be attributed to a portion of amorphous copper oxide being present. Additionally, in this sample, selenium appears to have a quite low concentration according to the latter techniques indicating the dominant role of DDT as a sulfur source, which was in excess and overshadowed the role of elemental Se as a precursor. In fact, the DDT can act as a surfactant, too, thus explaining the abundance of sulfur compared with selenium. In other words, a part of the sulfur may stay on the particle surface, rather than inserting the crystal lattice of the formed nanostructures. The UV-Vis-NIR spectrum of the light brown colloidal dispersion of this sample contains a major plasmon band at 1390 nm (Figure 8b). It has been suggested that the initial plasmon bands as soon as the particles are synthesized may be centered to higher wavelengths (e.g., 1700 nm), and oxidation is the driving force that provokes a blue-shift to ~1300 nm or lower wavelengths [18].

**Figure 9:**
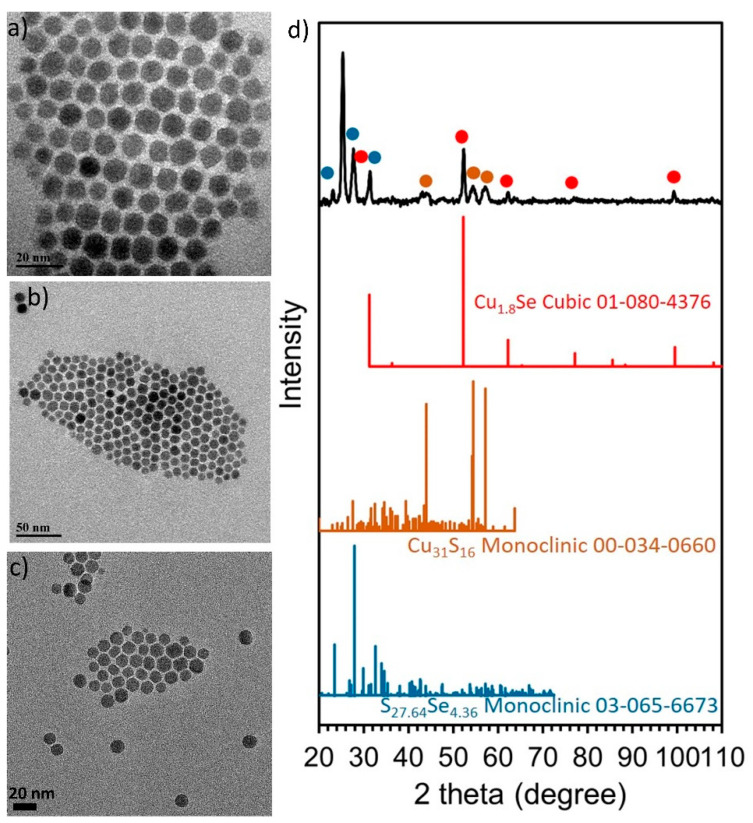
TEM images of sample Sa8 (**a**–**c**) and XRD measurement (**d**).

Finally, for the synthesis of the sample Sa8 we kept the same conditions as in the case of the sample Sa7, but we used the Cu(acac)_2_ precursor instead of Cu(OAc)_2_. Interestingly, despite the different reactivity between the two precursors, the size of the NPs of Sa8 was similar to that of Sa7, being measured at 8.8 ± 1.4 nm. A good assembly of the NPs onto the grid was shown as soon as the carrier solvent (toluene) evaporated (Figure 9a–c). It seems that the combination of reagents in Sa8 led to a good degree of size and shape monodispersity, also allowing a sufficient interparticle distance, thus resulting in that type of assembly. In fact, the XRD measurement of the Sa8 (Figure 9d) implies that this sample consists of both cubic Cu_1.8_Se and monoclinic Cu_31_S_16_ phases. The corresponding ICDD patterns of the above phases are the 01-080-4376 and 00-034-0660. The relative excess of sulfur in these particles, compared with selenium, as shown by ICP-AES (Cu:S:Se = 3.2:1:0.3) can be explained by the same reasons that were valid for sample Sa7, as mentioned previously. In particular, also in this case, the use of DDT resulted in the incorporation of sulfur in the crystal lattice of the product, but at the same time DDT can function as a capping agent, thus keeping an amount of sulfur on the surface of the particles. Quite clearly, visible crystalline domains are observed in representative particles at the HRTEM images of this sample (Figure S13). The mixed sample composition hinders a safe assignment for the measureable lattice planes to a specific component. STEM-EDX compositional mapping (Figure S14) demonstrates the homogeneous distribution of the different elements throughout the particle volume in this sample and is in agreement with the proportion between the different elements shown by ICP-AES and XRD. Regarding the UV-Vis-NIR spectrum of sample Sa8, a wide band at around 1600 nm is observed (Figure S15 at the Supplementary Materials).

### 2.2. Tauc Plots—Band Gaps

Copper selenide presents direct and indirect transitions, and the corresponding band gaps range between 2–3 eV and 1.1–1.5 eV, respectively. In the nanoscale, such values can reach 4 eV and 1.87 eV for direct and indirect transitions [4,42]. Copper sulfide has a band gap in the range 1.2–2 eV for its bulk form [43,44], with certain stoichiometries displaying indirect transitions, whereas others possess direct transitions [45,46].

Sample Sa1 has a direct band gap of 2.58 eV and an indirect one of 1.58 eV, as shown in Figure 1. The corresponding values for Sa2 are 2.79 eV and 1.93 eV, as depicted in Figure S16. The increase in the optical band gap can be assigned to the size decrease in Sa2, compared with Sa1 [47]. However, the band gap deduced for Sa2 denotes that this sample may contain a significant portion of copper selenide phase, since such values are rather unusual for copper sulfide compositions. The slightly smaller average size of Sa3 is in agreement with its higher values of direct and indirect E_g_ (2.86 and 2.00 eV, respectively, Figure S17). On the other hand, the somewhat bigger mean size of Sa4 (11.2 nm) does not seem to cause considerably different band gap values (2.83 eV and 2.29 eV for direct and indirect transitions, Figure S18). Additionally, in this sample, these values imply the presence of a significant amount of copper selenide phase, as mentioned above for Sa2. Moreover, the quite small size of Sa5 (~5 nm) is in agreement with the increased E_g_ values (2.92 and 2.35 eV for the two transition types, respectively; Figure S19). A considerable portion of copper selenide phases seems to be present also in this sample, judging by the band gap values, apart from copper sulfide compositions. The large nanoplate size and nearly stoichiometric Cu-Se composition in sample Sa6 caused lower E_g_ values (2.35 eV and 1.45 eV for direct and indirect transitions, Figure S20). A trend to decrease the direct band gap when comparing Cu_2_Se NPs and CuSe nanoplates has also been observed in a report by Zha and co-workers [48]. Finally, samples Sa7 and Sa8 have similar sizes, and their band gap values are only slightly different (see Figures S21 and S22). Variations in the Cu/S/Se proportions between the two samples can explain those small differences, especially in the direct band gap value (2.72 vs. 2.88 eV). The relation of band gaps with the electrocatalytic activity is presented in the section below.

### 2.3. Nitrogen Reduction Reaction

Ammonia synthesis under environmentally benign conditions is currently pursued, considering the importance of NH_3_ as a widely used chemical reagent in several industrial sectors. Though noble metal electrocatalysts have been frequently proven as very efficient for the NRR, their scarcity and high price make a scaled-up process practically not feasible. Inspired by a review paper by Zhou et al., who describe that Cu-based electrocatalysts can display a promising performance for the NRR, we decided to evaluate such activity for some of our samples [49]. The high abundance, relatively low cost and transition metal electronic structure make such investigations for their NRR capacity worth to work on [49].

In addition, metal chalcogenides such as MoS_2_ and MoSe_2_ nanosheets have displayed remarkable performance in the NRR with competitive Faradaic efficiencies. The presence of chalcogen atoms and defects such as vacancies has been reported to be beneficial for the NRR. In particular, chalcogen defects may lead to a more spontaneous and exothermic N_2_ adsorption step, whereas chalcogen sites can improve the energy profile of the reaction by donating protons for the first protonation of N_2_ [50]. Apart from Mo-based electrocatalysts, other dichalcogenides such as TiS_2_ and NbS_2_ nanosheets have been reported as efficient materials for the NRR [46]. With all the above in mind regarding Cu-containing and chalcogen-containing compounds, we decided to investigate the NRR activity of some of our copper- and chalcogenide-containing samples. NRR experiments were performed using samples which have been left outside the glovebox for a prolonged time (more than 12 months), thus any surface oxide layers that might have been formed are expected to passivate the nanoparticles core, hindering further oxidation and offering compositional stability. Samples Sa7, Sa8 were tested first due to their relatively small and monodisperse size, probably allowing a high surface area, suitable for catalysis. A CV was recorded first for Sa7 between 0.2 and −2 V in 0.1 M KOH (pH = 13), where two pairs of redox peaks were noticed at −0.4, −0.17, −1.5 and −1 V (Figure S23). The anodic peaks could be attributed to the oxidation of copper chalcogenide phases (e.g., Cu_2_Se to CuSe (Cu^+^ → Cu^2+^ + e^–^) or to the formation of electrochemically generated Cu-oxohydroxides), whereas cathodic peaks may be assigned to the reduction in copper chalcogenide phases (e.g., CuSe to Cu_2_Se (Cu^2+^ + e^–^ → Cu^+^) or to the reduction in the sorbed copper sulfide phase) [51,52]. The respective CV recordings for samples Sa8 and Sa6 are depicted at Figures S24 and S25 of the Supplementary Materials.

The NRR activity of sample Sa7 was evaluated in the same electrolyte in an H-cell at potentials of −1.8 and −1.9 V. The highest ammonia yield was observed at −1.9 V, as demonstrated, producing 115 ± 0.1 μgh^–1^ cm^–2^, corresponding, however, to a limited Faradaic efficiency of 0.38 ± 0.1%. In order to achieve the best possible accuracy and reliability of the data derived, three independent measurements were taken each time with a clean GC electrode under similar conditions. Interestingly, when applying the potential of −1.8 V, a Faradaic efficiency (FE) of 1.97 ± 0.2% was derived, even though the NH_3_ yield was at 88.2 ± 0.1 μgh^−1^ cm^−2^. More detailed results are shown in Table 1.

The NRR performance of sample Sa8 was then studied: more specifically, the highest FE for Sa8 was observed at a potential of −1.7 V (FE: 2.1%) with an ammonia yield of 112 ± 0.1 μgh^–1^ cm^–2^. The use of a −1.8 V potential resulted in a similar value for ammonia yield whereas the FE decreased to 1.03%. Raising the working potential to −1.9 V led to an NH_3_ yield of 205 ± 0.1 μgh^–1^cm^–2^ but with a quite low FE at 0.22% due to higher electrode charge.

The peculiar morphology of sample Sa6, with its very thin nanoplates, as shown by TEM and AFM measurements, possessing a high surface area and a good number of edges and sharp tips, motivated us to evaluate its electrocatalytic behaviour in NRR, too. Such morphological features are well known to favour high catalytic and electrocatalytic activities. Applying a potential of −1.9 V resulted in an ammonia yield of 190 ± 0.1 μgh^−1^cm^−2^, but with a low FE at 0.1%. The use of a −1.8 V potential provided a NH_3_ yield of 169 μgh^−1^cm^−2^ with a slightly higher Faradaic efficiency of 0.34%. An improved FE (1.29%) was observed when applying a potential of −1.7 V, with an ammonia yield of 174 μgh^−1^cm^−2^. Interestingly, lowering the applied potential further to −1.5 V led to a decrease for the ammonia yield at 99 μgh^−1^cm^−2^, but the FE was drastically improved to 8.8%.

**Figure 10:**
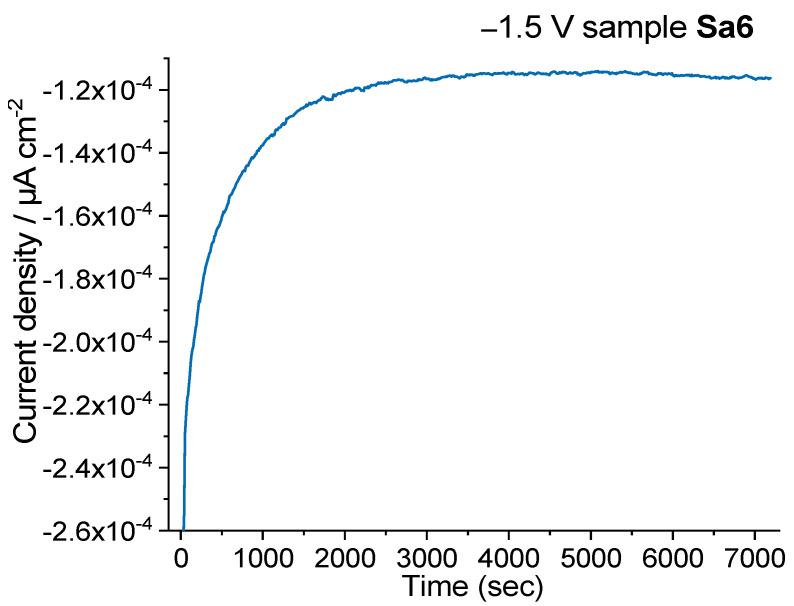
Chronoamperometry measurements for sample **Sa6** recorded over the course of a 2 h nitrogen reduction reaction using a potential of −1.5 V.

The stability of samples Sa7 and Sa8 toward the NRR was examined over the course of the two-hour reduction reaction where the potential was kept stable at a given value. In particular, for sample Sa7 one can observe a rather stable pattern for the current density, with no significant decrease during the NRR at a potential of −1.8 V, where it showed its highest FE (Figure S26). Similarly, when applying a potential of −1.7 V, there was no important decline for the current density upon the NRR two-hour slot with sample Sa8 as catalyst (Figure S27). The use of a −1.5 V potential for sample Sa6 demonstrated the stability of the latter sample, too, during a 2 h NRR interval (Figure 10). It seems that selecting a suitable potential was critical for acquiring an optimal value for NH_3_ yield and corresponding Faradaic efficiency. Then, the optimum FE values should be accompanied also by a stable behaviour for the materials under study.

The electrocatalytic NRR performance for samples Sa7 and Sa8 was comparable with various Cu-based electrocatalyst materials reported so far, such as Pd_3_Cu_1_ alloy, RhCu-BUNNs and CuO/RGO [49]. The corresponding activity of the thin 2D-nanoplates of sample Sa6 was even better, at least under certain circumstances, namely applied potentials. The intrinsic catalytic ability of copper also helps in this context. Overall, the relatively similar electrochemically active surface area (ECSA) values for Sa6, Sa7 and Sa8 (Figure S28) indicate similar populations of active sites, thus the adsorption and transfer of reactants to perform the NRR follows nearly common patterns in terms of speed. Possibly the capping ligands used during the synthesis of Sa6 deteriorated the electrocatalytic activity of the sharp tips and edges of its anisotropic structure by complicating the undisturbed access of reactants on these active sites [53]. Similar FE values have been obtained also for metal dichalcogenide materials with graphene-like structures such as c-MoS_2_ nanosheets grown on carbon cloth by hydrothermal method or MoSe_2_-based composites [50,54]. Chalcogens such as sulfur and selenium boost the NRR activity of the catalysts by acting as proton donors to the adsorbed dinitrogen and stabilizing the protonated reaction intermediates [54].

## 3. Conclusions

Copper selenide-sulfide nanostructures were prepared using one-pot heat-up protocols in the presence of a range of different precursors. For the first time it was shown that Al_2_Se_3_ can be used as aluminium source for the preparation of the nanoparticle system under discussion. The choice of the reaction conditions and especially the reactivity of the reagents affected not only the morphology of the resulting particles, but also their composition. In particular, the Cu_2−x_Se composition was favoured when no sulfur-containing compound was used, especially in the presence of aluminium selenide precursor and TOP. The use of TOPO promoted the acquisition of larger CuSe nanostructures with stoichiometric composition. On the other hand, adding DDT in combination with TOP and elemental Se was mostly associated with the acquisition of mixed copper selenide–sulfide compositions. However, it is highlighted that the plethora of possible copper chalcogenide crystal phases and compositions makes their precise identification quite complicated: a combination of different and complementary characterization techniques is more than necessary in order to shed a better light on the structural features of such nanostructures. The variation in band gap energies is assigned to the range of different Cu/S/Se proportions and crystal structures in the final nanostructures, as well as morphology and oxidation effects.

Even though the NH_3_ yield and FE of the copper chalcogenide nanostructures of our investigation may not yet meet levels which allow scale up and technological exploitation, they are deemed as promising for further studies, due to the low cost and high abundance of Cu. Better control of the particle shape, stability, surfactant coating, creation of more catalytically active sites, careful generation of defects and suppression of the parasitic hydrogen evolution reaction (HER) are approaches that will allow a more attractive catalytic performance with bigger selectivity for the NRR. Of course, other combinations of Cu- or chalcogen-based compositions will be also tested, aiming to optimize the NRR performance of such types of nanostructures.

## 4. Materials and Methods

Chloroform (99.8%), trioctylphosphine (90%), trioctylphospine oxide (90%), selenium powder (99.99%), lithium bis(trimethylsilyl) amide (LiN(SiMe_3_)_2_, 97%), 1-dodecanethiol (98%), oleylamine (80–90%), copper (II) acetylacetonate and copper (II) acetate were bought from Sigma-Aldrich (E. U.) Oleylamine (80–90%) was purchased from Acros Organics (E. U.) Oleic acid (99%), aluminium selenide (99%) and toluene (99%) were provided from Alfa-Aesar (E. U.). Ethanol (100%) was bought from HaymanKimia Ltd (UK). All reagents were used as received without additional purification.

### 4.1. Synthesis of CuSe Nanostructures

All synthesis stages were run using standard Schlenk techniques (Ar-filled glovebox, vacuum line, Schlenk tubes). This ensured inert gas conditions throughout synthesis and storage of reactants and products. For the synthesis of sample Sa1, 0.2 mmol of Cu(acac)_2_ and 20 mg of Al_2_Se_3_ were introduced in a three-neck flask containing 8.1 g OAm and 1.8 g oleic acid, in the presence of 33.5 mg of LiN(SiMe_3_)_2_. The mixture was degassed first at room temperature under stirring, performing three vacuum-N_2_ cycles for several minutes. After that, heating to 200 °C was applied at a rate of ~8 °C/min and the reaction flask was kept at that temperature for 45 min under N_2_ flow. Then, the mixture was left to cool down naturally at room temperature. The particles were collected by centrifugation with chloroform/ethanol (1:1 volume ratio) twice at 6000 rpm for 10 min each time. Eventually the nanoscale product was stored in toluene to give a light green solution.

For sample Sa2, there are similarities to the strategy used for the sample Sa1, but it was also partially inspired from our previous work on the CuTe system [1]. More specifically, we first mixed 0.6 mL OAm with 0.6 mL DDT at room temperature in a vial and then 95 mg of elemental Se were added. Stirring and degassing were applied as above. The resulting solution was added in a three-neck flask which contained 10 g OAm, 1.8 g oleic acid, 5 g TOP, 200 mg LiN(SiMe_3_)_2_ and finally, 240 mg Cu(OAc)_2_ (the insertion of those reagents was carried out following this sequence). Further degassing and heating took place as written above, with the same heating rate, but the final temperature was 200 °C. The mixture was held at this temperature for 20 min. Cooling, washing and storage of the nanoparticles were carried out as above, resulting in a dark brown colloidal dispersion.

The synthesis of sample Sa3 was as the one of Sa2, but the selenium source was not elemental Se, it was Al_2_Se_3_. Its amount was 116.3 mg. All the other reaction conditions, including final temperature and heating rate, were unchanged.

The sample Sa4 was synthesized as the Sa2, but Cu(acac)_2_ was used (314 mg) instead of Cu(OAc)_2_. Low product yield was observed in this sample, which had a brownish colour.

The sample Sa5 was similar to Sa2, but the reaction time was increased to 30 min and the temperature was 220 °C, aiming to somewhat increase the reaction yield. However, such yield visibly remained relatively low, whereas a considerable number of particles was lost also during washing stage. The final dispersion was brownish.

For sample Sa6, its synthesis was similar to that for Sa2, but the TOP was replaced by 1 g of TOPO. Pursuing a better solubility for elemental Se, the amounts of OAm and oleic acid were doubled in respect to sample Sa2. The produced nanoplates had a dark brown colloidal colour.

The protocol used for sample Sa7 was inspired and modified from [18] but it was also co-inspired by our own published approach for the synthesis of CuTe [1]. In particular, 8 mL DDT were mixed with 16 mL oleic acid and 240 mg Cu(OAc)_2_ were added, followed by 95 mg of elemental Se. After performing degassing cycles at 80 °C and re-cooling to room temperature, 3 mL TOP were added, followed by 3 mL OAm and 200 mg LiN(SiMe_3_)_2_. After further degassing at room temperature, the mixture was heated to 220 °C at a rate of 8 °C/min and it was kept at that temperature for 30 min. Cooling, washing and storage steps were the same as above, to give a dark brown colloidal dispersion.

Sample Sa8 was produced as Sa7, but the Cu(OAc)_2_ precursor was replaced by 314 mg of Cu(acac)_2_.

### 4.2. Characterisation Instruments

The nanoparticles were observed by transmission electron microscopy (TEM) using a JEOL JEM 1200-EX operating at an accelerating voltage of 120 kV with a Gatan DigitalMicrograph software). Colloidal dispersions of the samples were deposited on carbon-coated Cu grids and left to dry in air. Nickel-coated or gold-coated grids were used for the compositional quantification of the samples by HAADF-STEM-EDX (see below). Further imaging and compositional analysis were obtained with a FEI Tecnai G2 F20 S/TEM equipped with an EDX detection system. HAADF-STEM-EDX elemental mapping images were acquired with a JEOL 2200 FS microscope operating at an acceleration voltage of 200 kV. Elemental maps and EDS spectra were acquired with SDD detector X-MaxN 80 TS from Oxford Instruments (UK). Additional morphological characterization was performed with a FEI Magellan 400L HRSEM equipped with a through-the-lens detector (TLD). To obtain X-ray diffraction patterns, colloidal dispersions were dried to obtain powder samples for measuring at room temperature between 20–110°. A PANalytical X’Pert^3^ with Co source (λ = 1.789 Å) in transmission-reflection spinning mode was utilized for XRD measurements. Optical characterization was carried out with an ultraviolet-visible-near-infrared (UV-Vis-NIR) Carry 5000 spectrophometer using colloidal copper selenide dispersions in toluene. The concentrations of different elements present at the NPs was determined by inductively coupled plasma mass spectrometry (ICP). Powder samples were dissolved in concentrated nitric acid at 60 °C. Then, the solutions were diluted with deionized water to obtain a 2% nitric acid solution for elements quantification with a Varian 720 ICP-AES (Agilent). Atomic force microscopy (AFM) measurements were performed on a Ntegra Spectra (NT-MDT). Surface scans were carried out using a tapping (semi-contact) mode. Cantilevers with a strain constant of 1.5 kN m^−1^ equipped with a standard silicon tip with curvature radius lower than 10 nm were used for all measurements. AFM imaging was obtained by testing a drop-casted sample suspension (1 mg mL^−1^) on freshly cleaved mica substrate. The measurement was recorded in ambient conditions with a scan rate of 1 Hz and scan line of 512. The acquired data were processed with the software package Gwyddion.

Electrochemical experiments were implemented with an Autolab PGSTAT 204 (Metrohm, Switzerland) potentiostat. In the beginning, the glassy carbon (GC) electrode was polished with an alumina suspension to renew the electrode surface and then washed and wiped before the electrochemical reactions. The clean GC electrode was modified by depositing on its surface a 1.5 μL aliquot of the suspension of the material in study in acetonitrile and left to dry to generate a layer of homogeneously dispersed material. The original and modified GC electrodes were utilized as the working electrodes together with a KCl-saturated Ag/AgCl reference electrode and a platinum counter electrode.

The volume of the electrolyte (0.1 M potassium hydroxide) in the anode and the cathode was 50 mL for each, and a 35 mL acid trap of 0.1 M sulfuric acid was linked to both chambers. Both the anode and the cathode were degassed with pure N_2_ (99.999%) with a flow of 20 mL min^−1^ throughout the whole electrolysis process. CV voltammograms were acquired using a scan rate of 100 mV s^−1^, to examine the voltage window for NRR. The reactions took place at room temperature, and all potentials are referenced against KCl saturated Ag/AgCl. The amounts of ammonia and hydrazine produced were determined using a multiparameter photometer (Hanna Instruments, Woonsocket, RI, USA) as described previously by Antonatos et al. [55]. The ammonia yield as well as the Faradaic efficiency were calculated by using the equations described therein [55].

To determine the ECSA, the samples were cycled in a 0.1 M KOH electrolyte from 0.2 V to −0.2 V vs. SCE (saturated calomel electrode). This region was identified by previous testing and showed no faradaic reactions. The measurements were performed with various scan rates (20, 40, 60, 80 and 100 mV s^−1^) and the capacitance was determined from the dependence of current density (from geometric area) on scan rate.

## Figures and Tables

**Table 1:** NH_3_ yields and corresponding Faradaic efficiencies derived at different potentials for three distinct samples (ammonia yield: green colour—FE: black colour. Units: μgh^−1^cm^−2^ and % correspondingly).

	−1.5 V	−1.7 V	−1.8 V	−1.9 V
Sample Sa6	99/8.8	174/1.29	167/0.34	190/0.1
Sample Sa7		88/1.97	115/0.38	
Sample Sa8		112/2.1	112/1.03	205/0.22

## Data Availability

The data presented in this study are available on request by the corresponding author.

## Supplementary Materials

The following are available online at https://www.mdpi.com/article/10.3390/nano11123369/s1: Table S1: List of samples produced and experimental conditions. Figure S1: Photographs of the colloidal dispersions of some representative samples produced within this study. Figure S2: HAADF-STEM image and corresponding EDS spectrum for Sa1. Figure S3: UV-Vis-NIR spectrum for Sa2. Figure S4: UV-Vis-NIR spectrum for Sa3. Figure S5: TEM image of Sa4. Figure S6: UV-Vis-NIR spectrum for Sa4. Figure S7: XRD measurement for Sa5. Figure S8: HRTEM images for Sa5. Figure S9: BF-STEM image, EDX spectrum and corresponding elemental maps for copper, oxygen, sulfur, selenium, silicon and phosphorus and elemental analysis quantification for Sa5. Figure S10: HAADF-STEM image (top left) and corresponding EDS spectrum (bottom left) for Sa6. The bottom left image was obtained by tilting the TEM holder at 65 deg, which allows to estimate the thickness of the nanoplates. The bottom right SEM image allows also to estimate the thickness of the nanoplates. Figure S11: UV-Vis-NIR spectrum for Sa6. Figure S12: HAADF-STEM image, EDX spectrum and corresponding elemental maps for carbon, copper, oxygen, sulfur, silicon, selenium and elemental analysis quantification for Sa7. Figure S13: HRTEM images for Sa8. Figure S14: BF-STEM image, EDX spectrum and corresponding elemental maps for copper, sulfur, oxygen, selenium, silicon and elemental analysis quantification for Sa8. Figure S15: UV-Vis-NIR spectrum for Sa8. Figure S16: Tauc plots for direct and indirect transitions for Sa2. Figure S17: Tauc plots for direct and indirect transitions for Sa3. Figure S18: Tauc plots for direct and indirect transitions for Sa4. Figure S19: Tauc plots for direct and indirect transitions for Sa5. Figure S20: Tauc plots for direct and indirect transitions for Sa6. Figure S21: Tauc plots for direct and indirect transitions for Sa7. Figure S22: Tauc plots for direct and indirect transitions for Sa8. Figure S23: Consecutive CV scans of the sample Sa7 in 0.1 M KOH. Figure S24: Consecutive CV scans of the sample Sa8 in 0.1 M KOH. Figure S25: Consecutive CV scans of the sample Sa6 in 0.1 M KOH. Figure S26: Chronoamperometry measurement for sample Sa7 obtained during a 2 h-nitrogen reduction reaction at a potential of −1.8 V. Figure S27: Chronoamperometry measurement for sample Sa8 recorded over the course of a 2 h nitrogen reduction reaction using a potential of −1.7 V. Figure S28: Determination of electrochemical double-layer capacitance (Cdl) of the samples using cyclic voltammetry scans in a non-faradaic region. Reference [56] is cited in the supplementary materials.
