# Supplementary material for: Heat-Up Colloidal Synthesis of Shape-Controlled Cu-Se-S Nanostructures—Role of Precursor and Surfactant Reactivity and Performance in N2 Electroreduction
Source: Nanomaterials (Basel). 2021 Dec 12;11(12):3369. doi: 10.3390/nano11123369 (PMC8707546; doi:10.3390/nano11123369)
Supplement: Supplementary file 1 [file nanomaterials-11-03369-s001.zip › nanomaterials-1455267-supplementary.pdf]

nanomaterials

Supplementary Materials

Heat-Up Colloidal Synthesis of Shape-Controlled Cu-Se-S Nanostructures—Role of Precursor and Surfactant Reactivity and Performance in N_2 Electroreduction

Stefanos Mourdikoudis [1,2,3,*], George Antonaropoulos [4,5], Nikolas Antonatos [3], Marcos Rosado [6], Liudmyla Storozhuk [1,2], Mari Takahashi [7], Shinya Maenosono [7], Jan Luxa [3], Zdeněk Sofer [3,*], Belén Ballesteros [6,*], Nguyen Thi Kim Thanh [1,2,*] and Alexandros Lappas [4,*]

[1] Biophysics Group, Department of Physics and Astronomy, University College London, London WC1E 6BT, UK; l.storozhuk@ucl.ac.uk
[2] UCL Healthcare Biomagnetics and Nanomaterials Laboratories, 21 Albemarle Street, London W1S 4BS, UK
[3] Department of Inorganic Chemistry, University of Chemistry and Technology Prague, Technicka 5, 16628 Prague 6, Czech Republic; nikolaoa@vscht.cz (N.A.); jan.luxa@vscht.cz (J.L.)
[4] Institute of Electronic Structure and Laser, Foundation for Research and Technology-Hellas, Vassilika Vouton, 71110 Heraklion, Greece; ganton@iesl.forth.gr
[5] Department of Chemistry, University of Crete, Voutes, 71003 Heraklion, Greece
[6] Catalan Institute of Nanoscience and Nanotechnology (ICN2), CSIC and the Barcelona Institute of Science and Technology, Campus UAB, Bellaterra, 08193 Barcelona, Spain; marcos.rosado@icn2.cat
[7] School of Materials Science, Japan Advanced Institute of Science and Technology, 1-1 Asahidai, Nomi, Ishikawa 923-1292, Japan; mari@jaist.ac.jp (M.T.); shinya@jaist.ac.jp (S.M.)
* Correspondence: mourdikt@vscht.cz (S.M.); soferz@vscht.cz (Z.S.); belen.ballesteros@icn2.cat (B.B.); ntk.thanh@ucl.ac.uk (N.T.K.T.); lappas@iesl.forth.gr (A.L.)

Table S1: Chemical reagents and reaction temperatures for the heat-up synthesis of Cu-S-Se nanostructures *.

Sample name	Precursors	Rest reagents (solvents, surfactants, reductants)	Temp./Time
Sa1	Cu(acac)$_2$, Al_2Se_3	OAm, OAc, LiN(SiMe$_3$)$_2$	200 °C / 45 min
Sa2	Cu(OAc)$_2$, Se	OAm, OAc, DDT, TOP, LiN(SiMe$_3$)$_2$	200 °C / 20 min
Sa3	Cu(OAc)$_2$, Al_2Se_3	OAm, OAc, DDT, TOP, LiN(SiMe$_3$)$_2$	200 °C / 20 min
Sa4	Cu(acac)$_2$, Se	OAm, OAc, DDT, TOP, LiN(SiMe$_3$)$_2$	200 °C / 20 min
Sa5	Cu(OAc)$_2$, Se	OAm, OAc, DDT, TOP, LiN(SiMe$_3$)$_2$	220 °C / 30 min
Sa6	Cu(OAc)$_2$, Se	OAm, OAc, DDT, TOPO, LiN(SiMe$_3$)$_2$	200 °C / 20 min
Sa7	Cu(OAc)$_2$, Se	OAm, OAc, DDT, TOP, LiN(SiMe$_3$)$_2$	220 °C / 30 min
Sa8	Cu(acac)$_2$, Se	OAm, OAc, DDT, TOP, LiN(SiMe$_3$)$_2$	220 °C / 30 min

* For the exact amounts of the reagents and processes followed refer to the experimental part in the main manuscript.

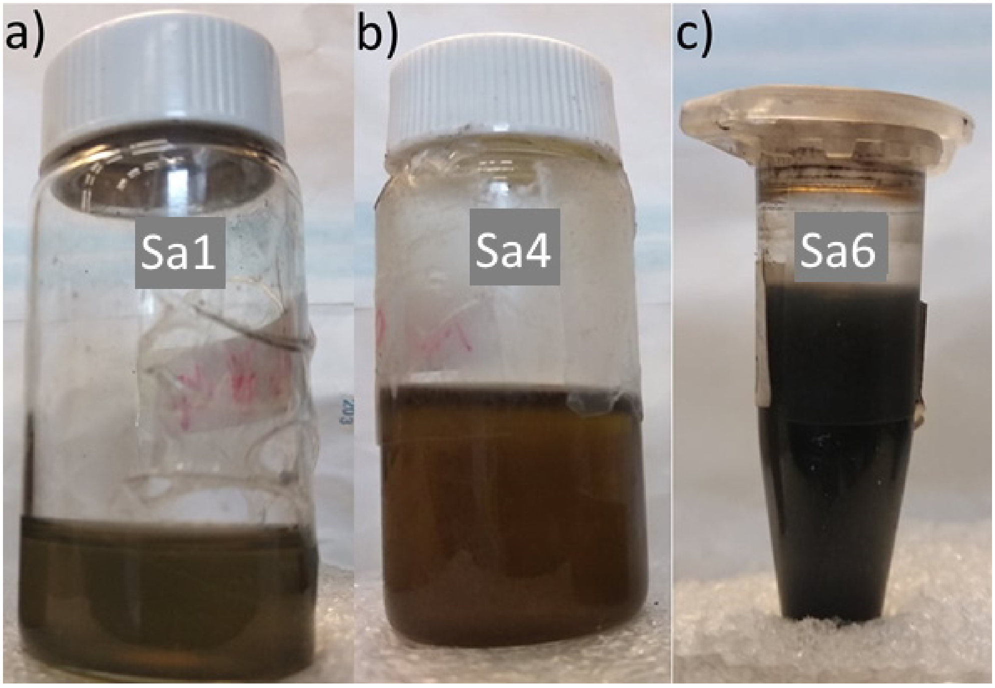

Figure S1. Photographs of the colloidal dispersions of some representative samples produced within this study.

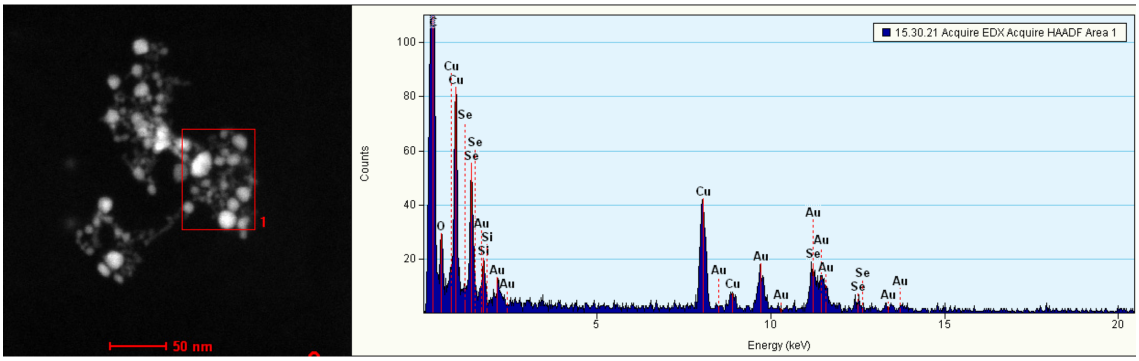

Figure S2. HAADF-STEM image (left) and corresponding EDS spectrum (right) for Sa1. The Au signal comes for the TEM grids used in this measurement.

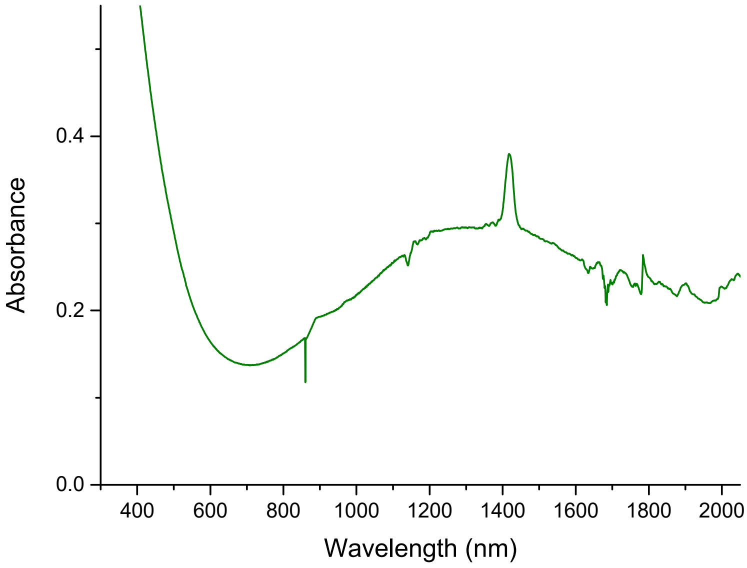

Figure S3. UV-Vis-NIR spectrum for Sa2.

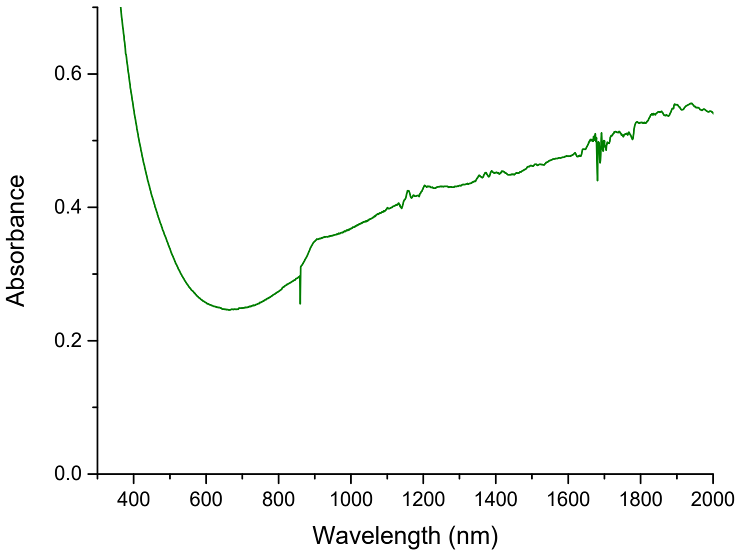

Figure S4. UV-Vis-NIR spectrum for Sa3.

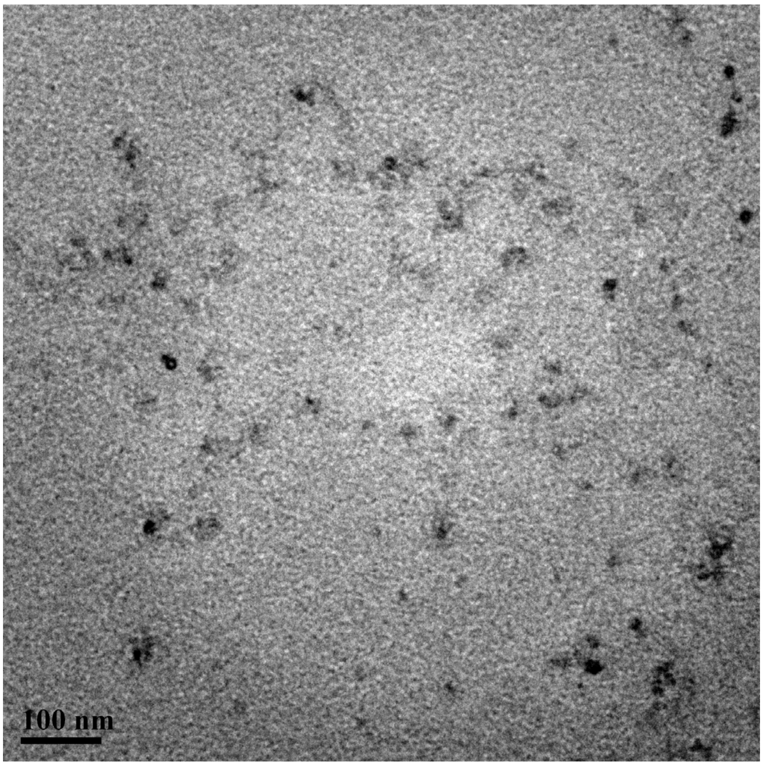

Figure S5. TEM image of Sa4.

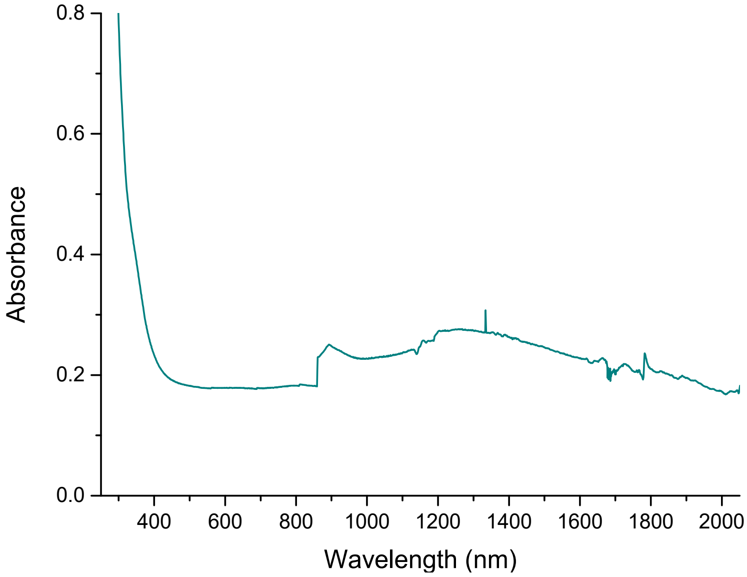

Figure S6. UV-Vis-NIR spectrum for Sa4.

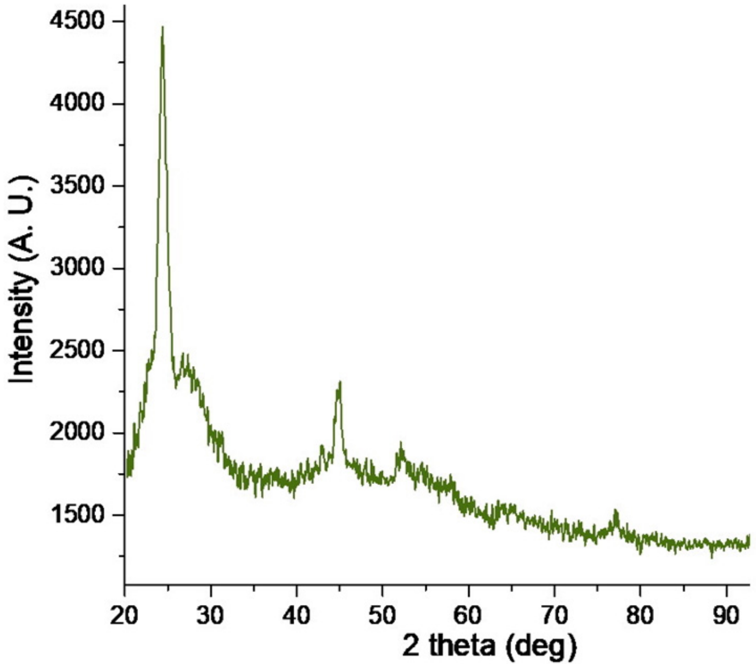

Figure S7. XRD measurement for Sa5.

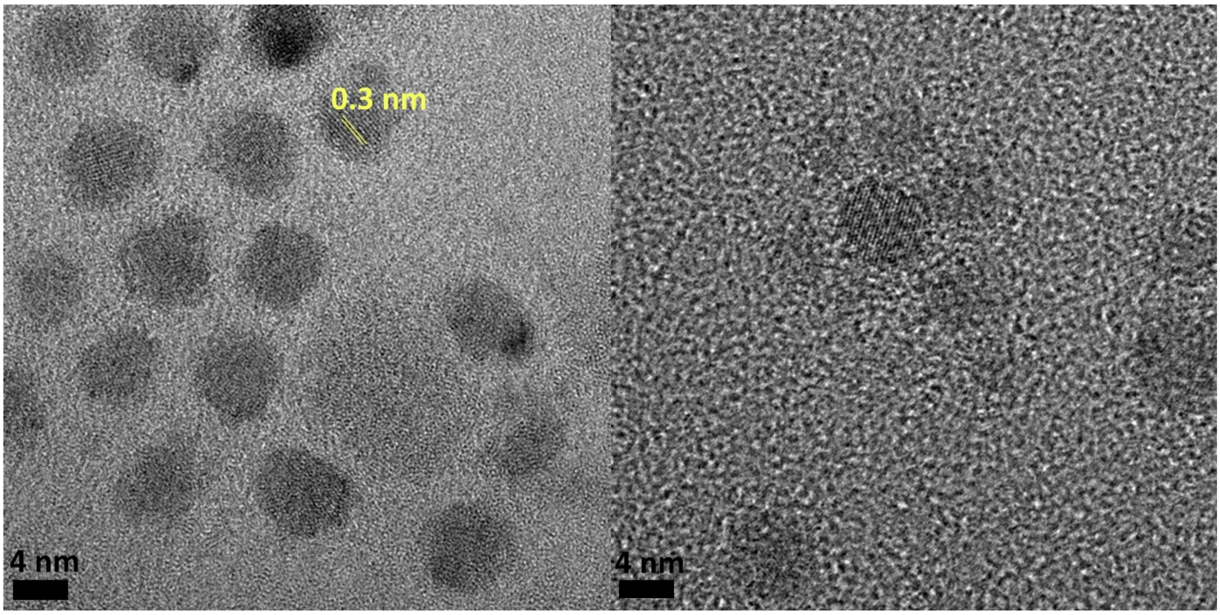

Figure S8. HRTEM images for Sa5.

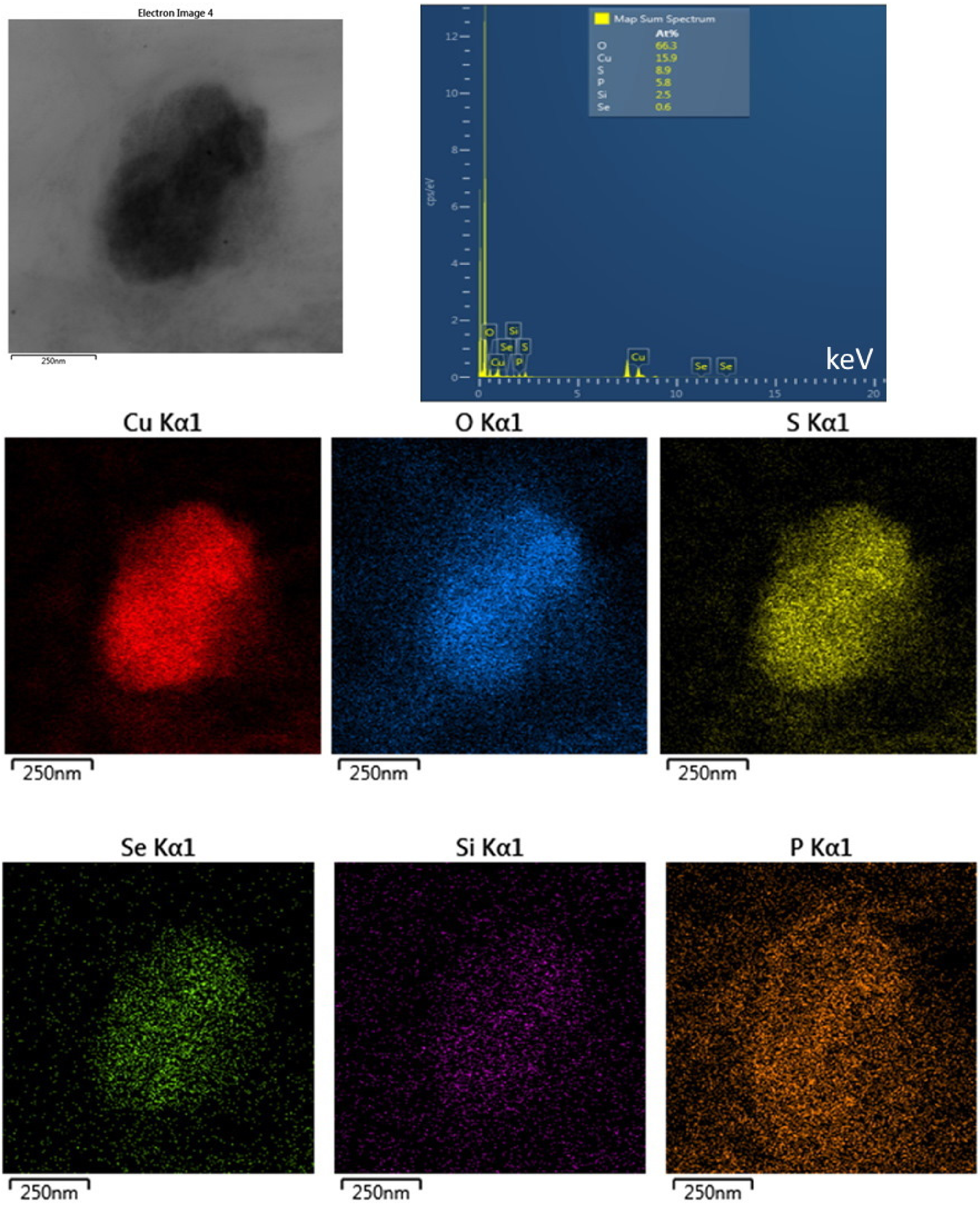

Figure S9. BF-STEM image, EDX spectrum and corresponding elemental maps for copper, oxygen, sulphur, selenium, silicon and phosphorus and elemental analysis quantification for Sa5.

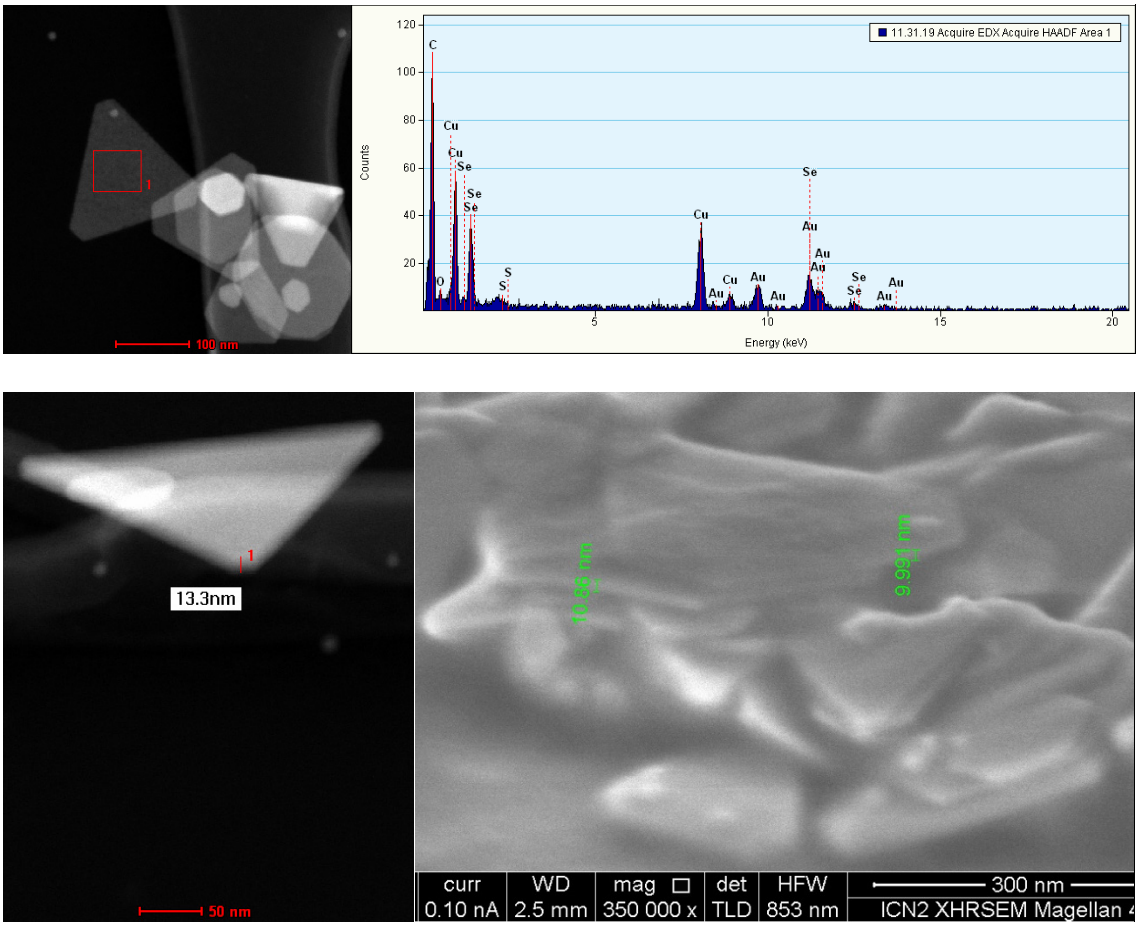

Figure S10. HAADF-STEM image (top left) and corresponding EDS spectrum (top right) for Sa6. The bottom left image was obtained by tilting the TEM holder at 65 deg, which allows to estimate the thickness of the nanoplates. The bottom right SEM image allows also to estimate the thickness of the nanoplates.

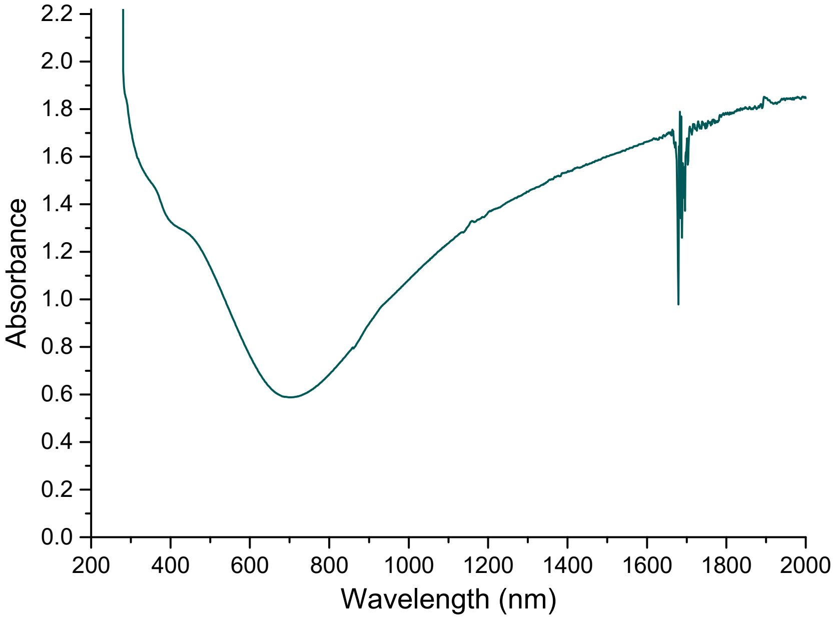

Figure S11. UV-Vis-NIR spectrum for Sa6.

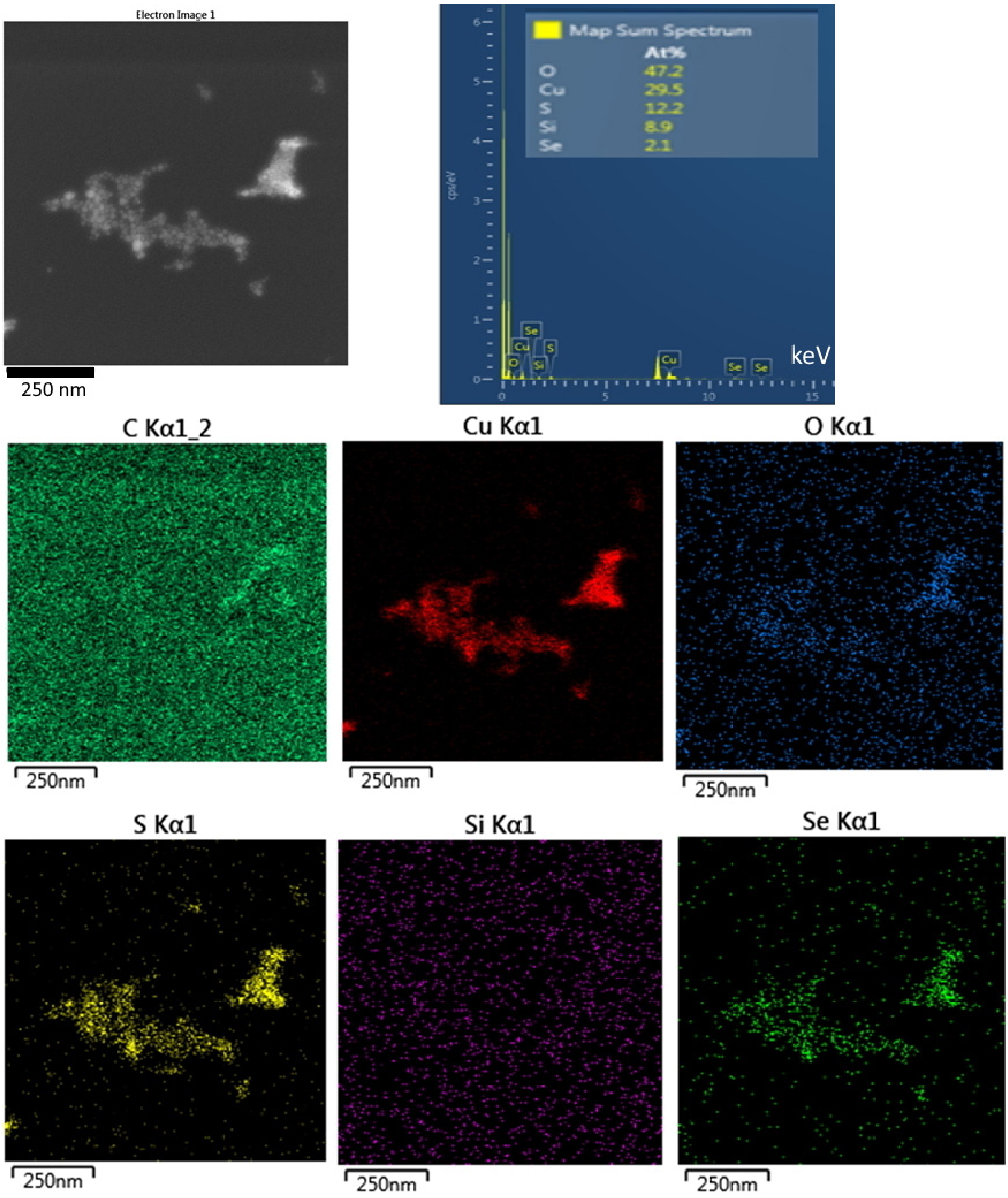

Figure S12. HAADF-STEM image, EDX spectrum and corresponding elemental maps for carbon, copper, oxygen, sulfur, silicon, selenium and elemental analysis quantification for Sa7.

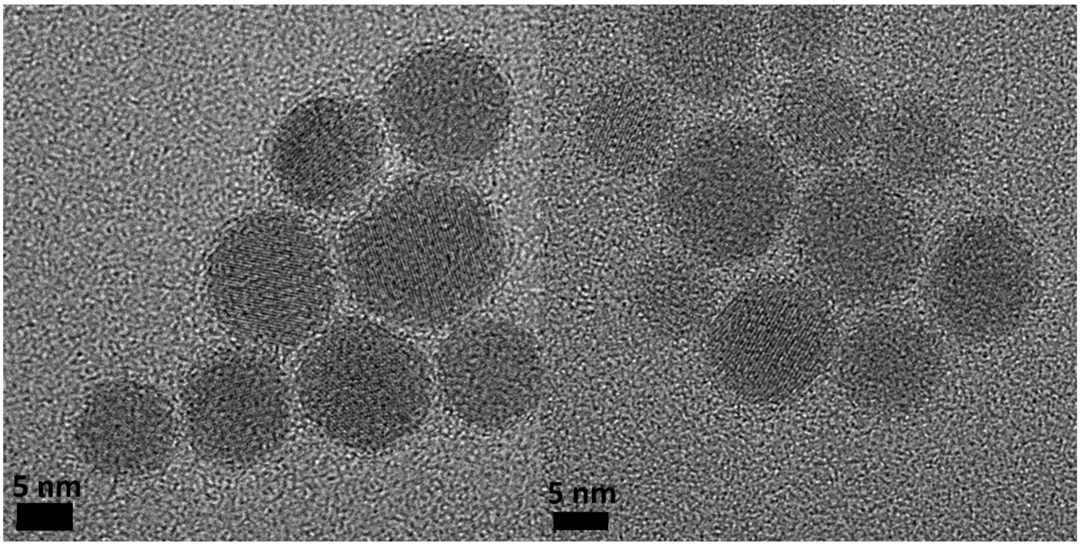

Figure S13. HRTEM images for Sa8

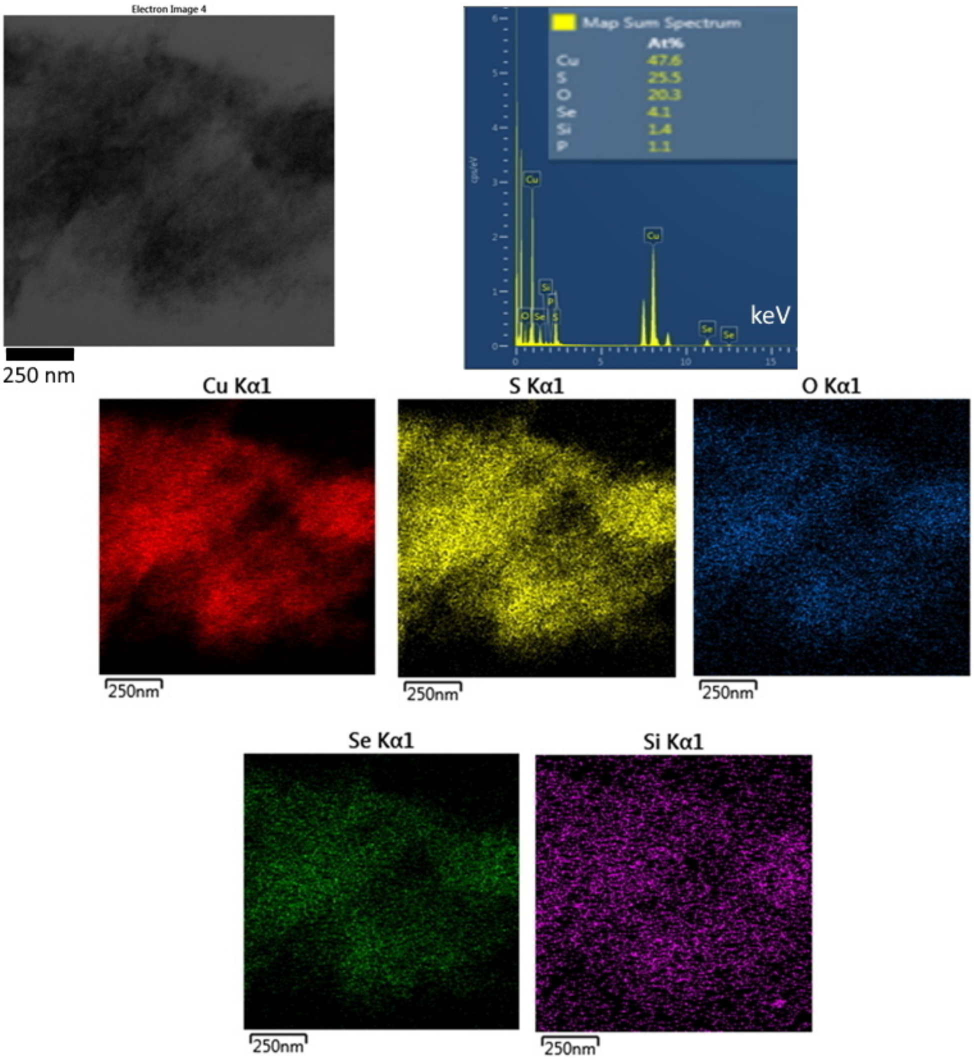

Figure S14. BF-STEM image, EDX spectrum and corresponding elemental maps for copper, sulfur, oxygen, selenium, silicon and elemental analysis quantification for Sa8.

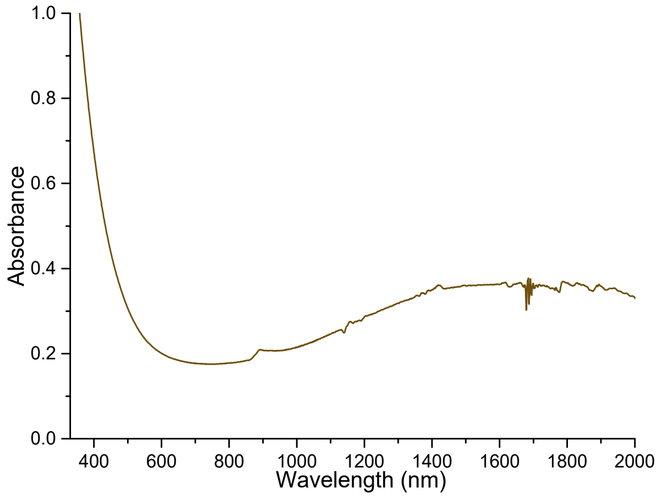

Figure S15. UV-Vis-NIR spectrum for Sa8.

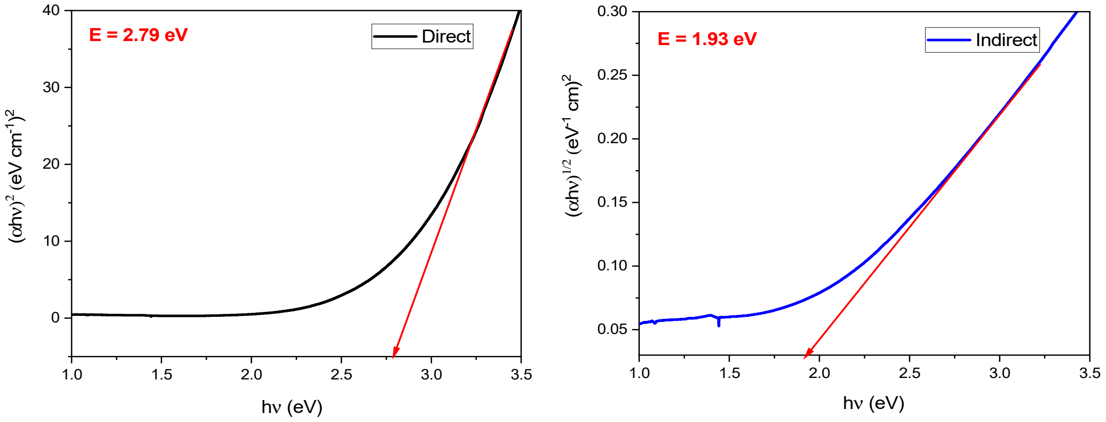

Figure S16. Tauc plots for direct and indirect transitions for Sa2.

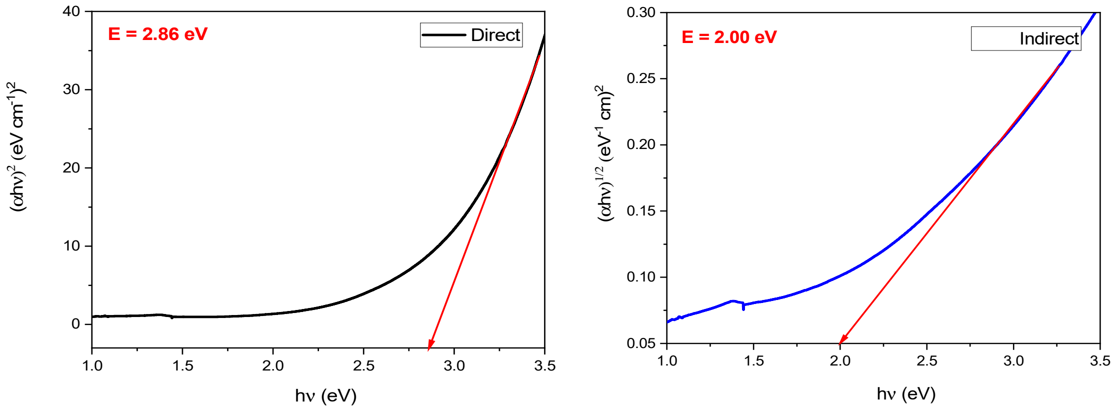

Figure S17. Tauc plots for direct and indirect transitions for Sa3.

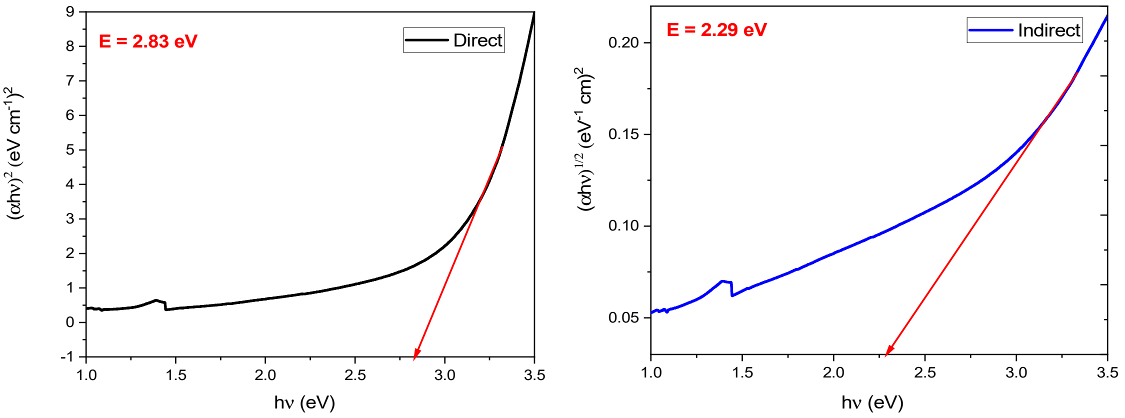

Figure S18. Tauc plots for direct and indirect transitions for Sa4.

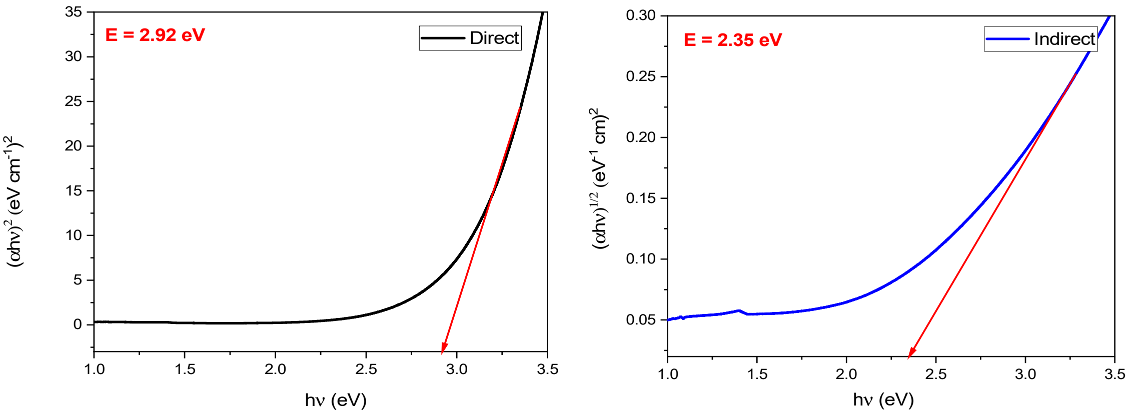

Figure S19. Tauc plots for direct and indirect transitions for Sa5.

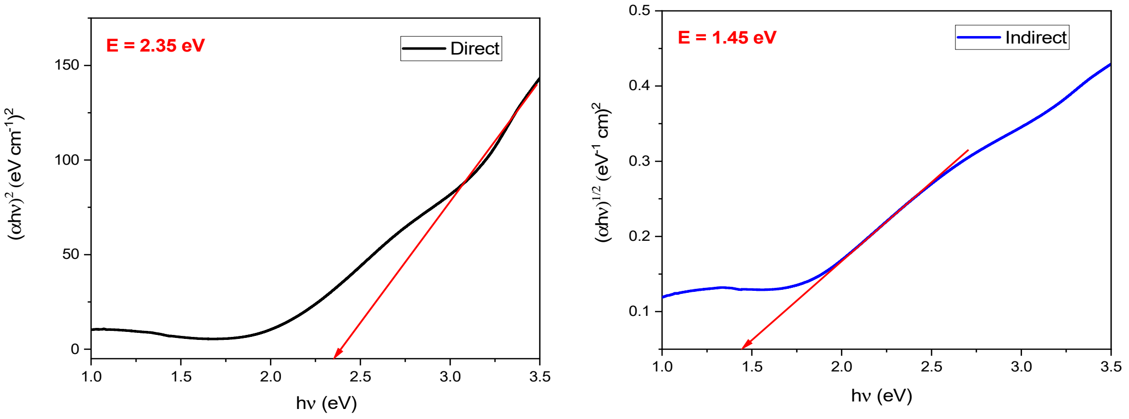

Figure S20. Tauc plots for direct and indirect transitions for Sa6.

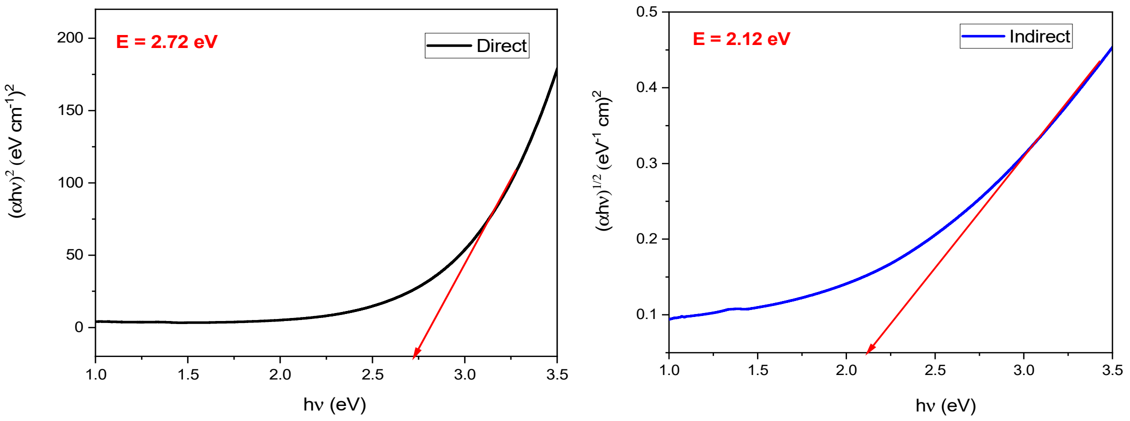

Figure S21. Tauc plots for direct and indirect transitions for Sa7.

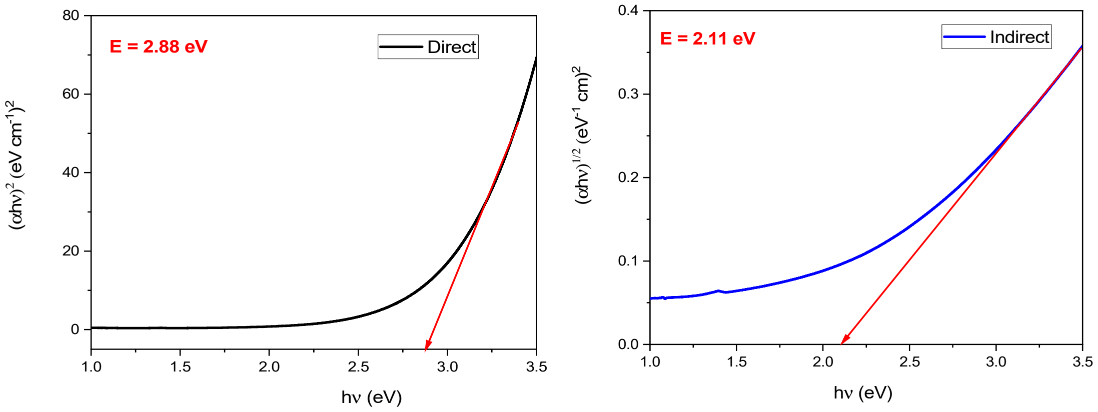

Figure S22. Tauc plots for direct and indirect transitions for Sa8.

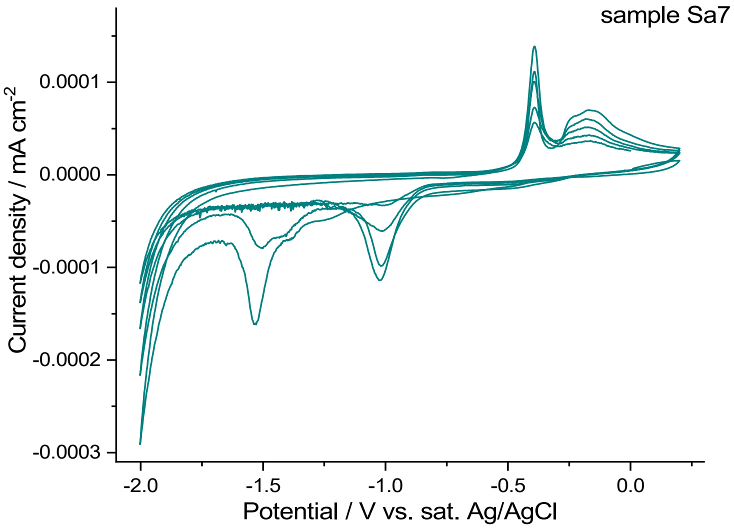

Figure S23. Consecutive CV scans of the sample Sa7 in 0.1 M KOH.

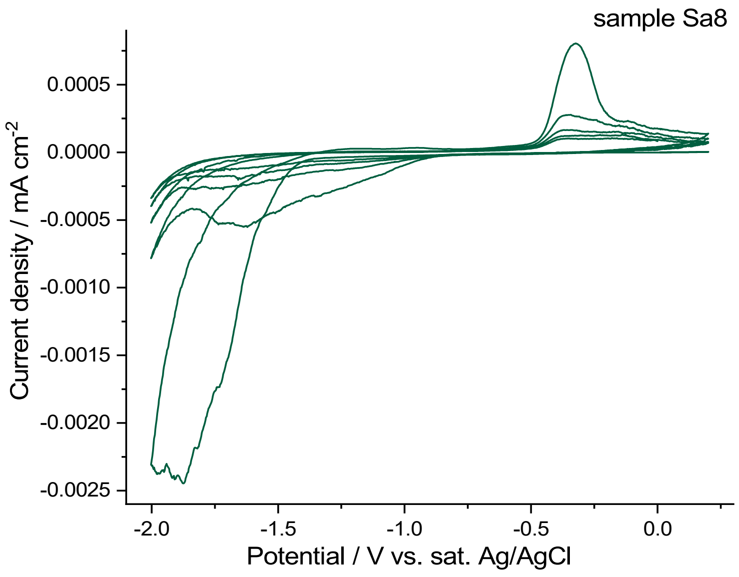

Figure S24. Consecutive CV scans of the sample Sa8 in 0.1 M KOH.

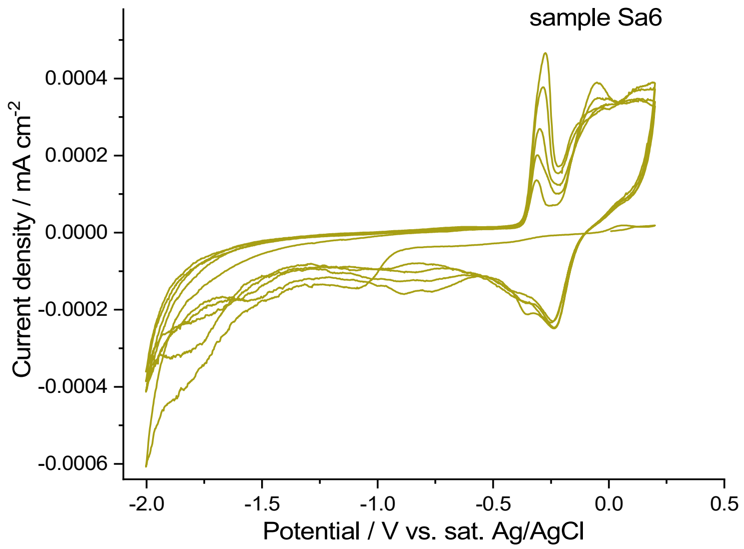

Figure S25. Consecutive CV scans of the sample Sa6 in 0.1 M KOH.

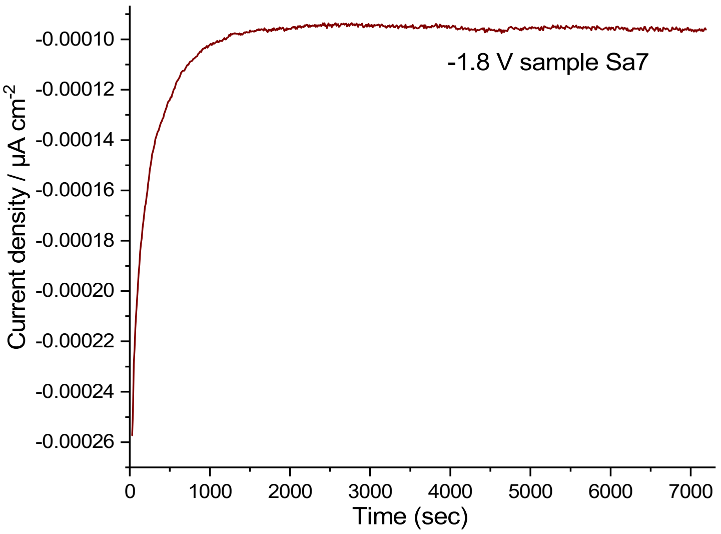

Figure S26. Chronoamperometry measurement for sample Sa7 obtained during a 2h-nitrogen reduction reaction at a potential of –1.8 V.

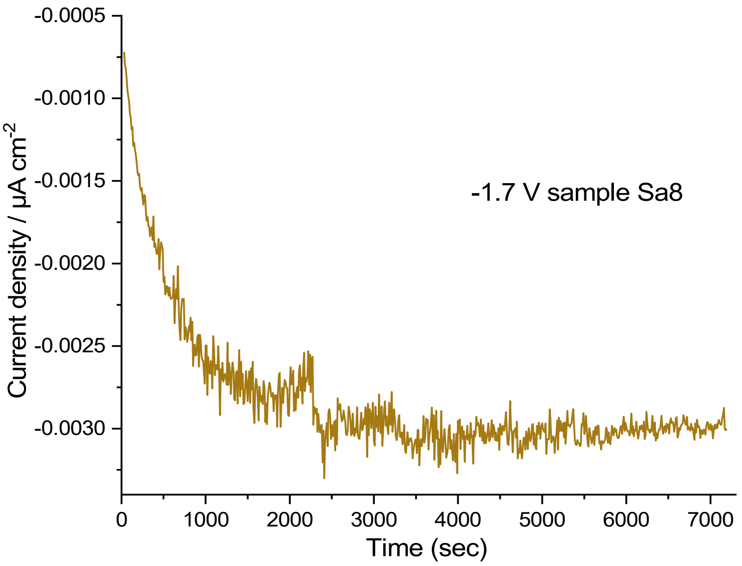

Figure S27. Chronoamperometry measurement for sample Sa8 recorded over the course of a 2h nitrogen reduction reaction using a potential of −1.7 V.

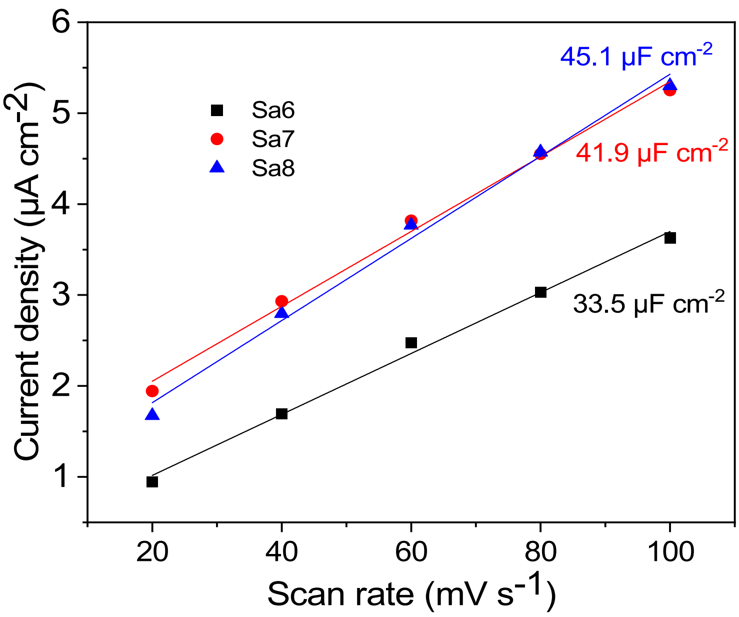

Figure S28. Determination of electrochemical double-layer capacitance (C_{dl}) of the samples using cyclic voltammetry scans in a non-faradaic region.

To extract the electrochemically active surface area we considered the capacitance of a flat surface as 20 μA.cm^{-2} [56]. After that the values of the ECSA were determined as 1.68 cm^2, 2.1 cm^2 and 2.26 cm^2 for Sa6, Sa7 and Sa8 respectively.
